# Iron Status in Patients with SARS-CoV-2 Infection

**DOI:** 10.3390/cimb48101062

**Published:** 2026-10-15

**Authors:** Bogdan Cylwik, Ewa Gruszewska, Magdalena Lozowicka, Anatol Panasiuk, Lech Chrostek

**Affiliations:** 1 Department of Pediatric Laboratory Diagnostics, Medical University of Bialystok, 15-274 Bialystok, Poland; 2 Department of Biochemical Diagnostics, Medical University of Bialystok, 15-269 Bialystok, Poland; ewa.gruszewska@umb.edu.pl (E.G.);; 3 Department of Pediatric Laboratory Diagnostics, University Children Clinical Hospital in Bialystok, 15-274 Bialystok, Poland; 4 Department of Gastroenterology, Hepatology and Internal Diseases, Voivodeship Hospital in Bialystok, 15-950 Białystok, Poland; 5 Department of Clinical Medicine, Medical University of Bialystok, 15-269 Bialystok, Poland

**Keywords:** iron status, transferrin, TSAT, sTfR, ferritin, hepcidin, COVID-19

## Abstract

This study evaluated the iron status and diagnostic usefulness of iron-related parameters in patients with SARS-CoV-2 infection upon admission to the hospital and during hospitalization. The study group included 87 patients with COVID-19 and 45 healthy subjects. Serum iron, ferritin, transferrin, transferrin saturation (TSAT), soluble transferrin receptor (sTfR), and hepcidin concentrations were measured using routine laboratory methods. Serum iron, transferrin concentrations, and TSAT were significantly decreased in COVID-19 on hospital admission, before initiation of treatment, whereas ferritin and hepcidin levels were increased in comparison to the control group (*p* < 0.001, *p* < 0.001, *p* = 0.048, *p* < 0.001, and *p* < 0.001, respectively). Serum iron and ferritin levels were significantly higher after hospitalization than those on admission (*p* < 0.05 for all comparisons). Significant differences were observed only in ferritin and transferrin levels between the groups categorized according to disease severity (*p* = 0.002 and *p* < 0.001, respectively); levels were significantly higher in patients with critical disease severity compared to those with moderate disease severity (*p* = 0.005 and *p* < 0.001, respectively). Transferrin also showed a negative correlation with disease severity (*p* = 0.040). Serum ferritin and TSAT levels were significantly higher, whereas transferrin concentration was significantly lower in patients with cytokine storm compared to those without it (*p* < 0.001, *p* = 0.011, and *p* < 0.001, respectively). Ferritin and TSAT also showed a positive correlation, while transferrin revealed a negative correlation with the cytokine storm (*p* < 0.001, *p* = 0.011, and *p* < 0.001, respectively). Additionally, serum ferritin concentrations were significantly decreased, while transferrin levels were increased in survivors compared to non-survivors (*p* = 0.015 and *p* = 0.001, respectively). Changes in ferritin, transferrin, TSAT, and hepcidin concentrations were significantly associated with inflammatory markers and hematological parameters. The results of our study indicate that iron homeostasis is disrupted in patients with COVID-19. This dysregulation may contribute to impaired hemoglobin synthesis and could play a role in the development of anemia of inflammation. Furthermore, alterations in iron-related biomarkers were associated with disease severity in patients with SARS-CoV-2 infection. While these findings suggest a potential role for these indices in disease assessment, additional studies incorporating appropriate predictive analyses are required to establish their diagnostic and prognostic value.

## 1. Introduction

Coronavirus disease 19 (COVID-19) is a contagious disease that is caused by severe acute respiratory syndrome coronavirus 2 (SARS-CoV-2). It appeared in December 2019 and remains a major global health challenge [1,2]. By June 2026, the cumulative total of confirmed COVID-19 cases reported to the WHO reached 779,220,015 (cumulative total), with approximately 1,115,793 deaths attributed to this disease [3]. Although primarily affecting the respiratory system, COVID-19 is increasingly recognized as a systemic disease characterized by immune dysregulation, hyperinflammation, endothelial dysfunction, and multiorgan involvement. Disease severity varies considerably among patients, ranging from asymptomatic infection to severe pneumonia, acute respiratory distress syndrome (ARDS), and death [1,2,4,5,6,7,8].

Iron is one of the most important trace elements necessary for the proper functioning of the body [9,10]. It is involved in numerous biological processes, including oxygen transport, erythropoiesis, mitochondrial function, cellular metabolism, and many enzymatic reactions. Furthermore, iron is essential for normal immune function and protection against viral and bacterial infections. It maintains the balance between the host defense system and virus proliferation and therefore plays an important role in infectious diseases. Disruption of this balance may contribute to abnormal immune response and increased susceptibility to infections. A close relationship has been demonstrated between the body’s current iron stores and the risk of acute infections, with both iron deficiency and iron overload increasing the risk of these infections [11,12,13,14,15,16,17,18]. During acute inflammatory conditions, proinflammatory cytokines, particularly interleukin-6 (IL-6), stimulate hepatic hepcidin production, which leads to decreased intestinal iron absorption and retention of iron within macrophages [19]. Consequently, circulating iron concentrations decline, while ferritin levels increase. Elevated ferritin concentrations have been consistently linked to disease severity, cytokine storm, and adverse clinical outcomes. Similar alterations have been reported in patients with COVID-19, suggesting a profound disturbance of iron metabolism associated with the inflammatory response [5]. During infectious and inflammatory states, iron metabolism undergoes profound and rapid alterations. This response limits the availability of circulating iron to invading pathogens but simultaneously reduces iron accessibility for erythropoiesis and cellular metabolism [11,12,13,14,15,16,17,18]. Due to the growing evidence of the significant role of iron in the body’s immune response, iron metabolism has become a subject of interest in the new coronavirus disease, COVID-19.

Several studies have demonstrated significant alterations in iron-related parameters, including serum iron, ferritin, transferrin, and other markers of iron homeostasis, in patients with COVID-19 [4,6,20,21,22,23,24,25,26,27,28,29]. These abnormalities have been proposed as potential indicators of disease severity and clinical outcome [20,21]. Growing evidence supports a link between SARS-CoV-2 infection and profound disturbances in iron homeostasis; nevertheless, the prognostic and pathophysiological significance of these changes remains unclear and incompletely understood. Moreover, published data are often inconsistent.

The aim of the study was to evaluate iron status in hospitalized patients with SARS-CoV-2 infection by measuring serum iron, ferritin, transferrin, transferrin saturation (TSAT), soluble transferrin receptor (sTfR), and hepcidin upon hospital admission and during hospitalization. Additionally, we explored the associations between iron-related tests and inflammatory parameters and COVID-19 severity to further elucidate the role of iron dysregulation in disease progression and to explore the potential diagnostic utility of iron-related laboratory tests as biomarkers of disease severity.

## 2. Materials and Methods

### 2.1. Patients

**Figure 1:**
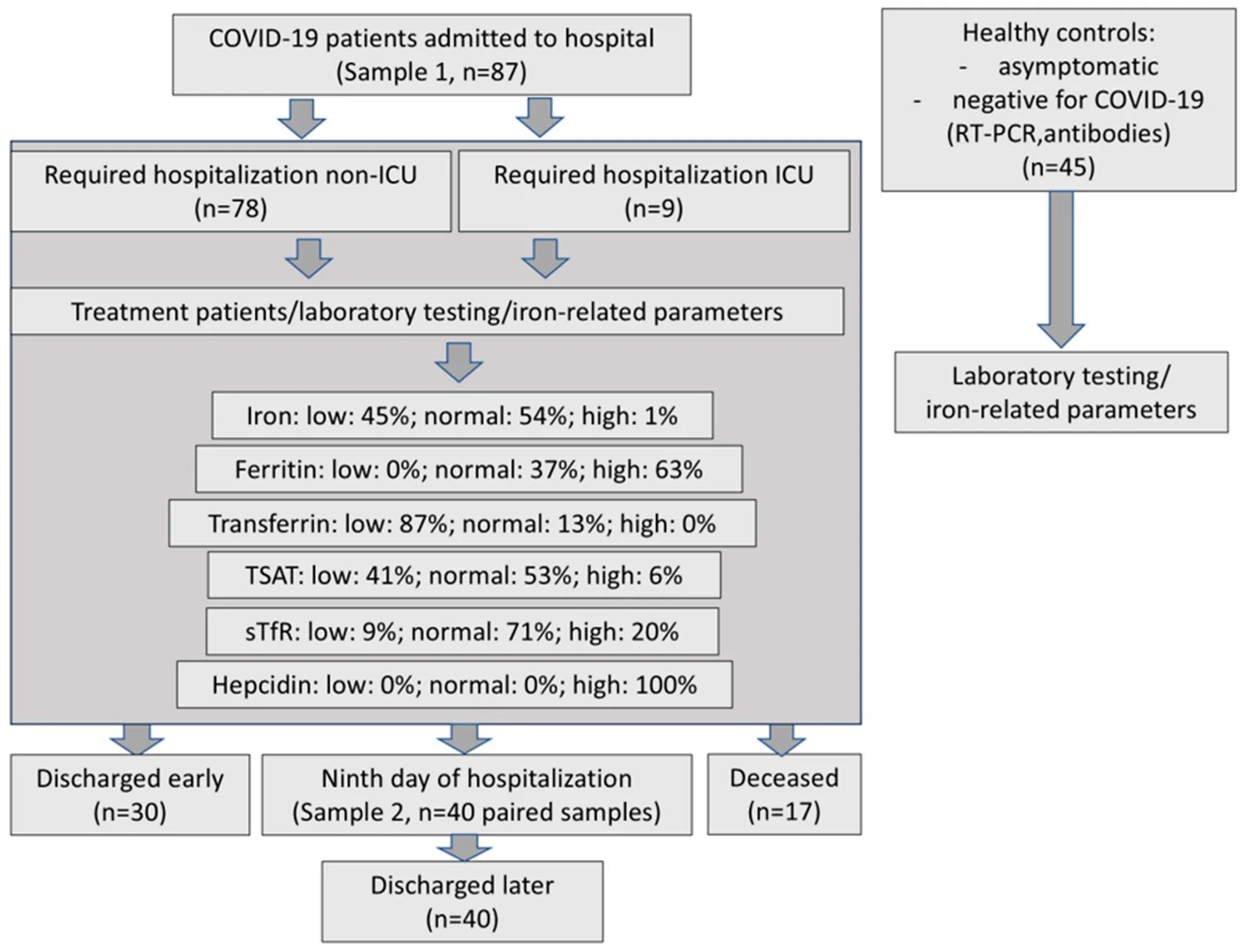
The flow chart of patient recruitment and the distribution of iron-related parameters in the study population.

The study group comprised 87 patients (33 females, 54 males; mean age 59.2 years, range 22–89) admitted to the Department of Gastroenterology, Hepatology and Internal Diseases with the Centre for Diagnostics and Endoscopic Treatment for COVID-19 between 20 November 2021 and 16 March 2022 (Figure 1). Initial screening for SARS-CoV-2 was performed using a rapid antigen test (Panbio^PM^ COVID-19 Ag) (Abbott Rapid Diagnostics Jena GmbH, Jena, Germany), with confirmation by RT-PCR (SARS-CoV-2 Triplex PCR) (Astra Biotech GmbH, Berlin, Germany). The patients were infected with the Omicron variant of SARS-CoV-2. All patients underwent routine laboratory (biochemical, hematological, and coagulation), arterial blood gas, CO-oximetry, and imaging assessments at admission.

Patients were classified into three categories according to disease severity assessed using the Modified Early Warning Score (MEWS): moderate (*n* = 58), severe (*n* = 13), and critical (*n* = 16). Moderate cases comprised symptomatic patients (fever, cough, anosmia, and lack of dyspnea) with evidence of lower respiratory tract involvement but no respiratory failure (SatO_2_ ≥ 94%) and only mild radiographic changes. Severe disease was defined by SatO_2_ < 94%, respiratory rate > 30 breaths/min, lung infiltrates involving more than 50% of the lung parenchyma, or PaO_2_/FiO_2_ ratio < 300 indicating respiratory failure and/or ARDS. Critical cases were characterized by respiratory failure/ARDS accompanied by multiorgan dysfunction (according to WHO, 2020) [30]. A Modified Early Warning Score (MEWS) was calculated on the basis of six parameters: systolic blood pressure, heart rate, respiratory rate, SatO_2_, body temperature, and AVPU score (alert, verbal, pain, and unconscious) [31]. Each of the MEWS components was ranked on a scale from 0 to 3 points based on the degree of parameter derangement (Table 1). The total score was the sum of each component. Moderate, severe, and critical patients scored <3, 3–4, and >4 points, respectively. Among the COVID-19 patients, 28 (32.2%) developed a cytokine storm, defined as interleukin-6 (IL-6) levels above 100 pg/mL, and 66 (75.9%) had chronic comorbidities. The most frequent conditions were hypertension (*n* = 21), type 2 diabetes mellitus (*n* = 15), and cirrhosis (*n* = 8). Based on patient-reported data, 29 individuals (33.3%) were vaccinated, whereas 55 were unvaccinated; among those vaccinated, 9 had received one dose, 12 two doses, and 8 three doses. Of the 87 patients included, 17 died during hospitalization (19.5%), 9 (10.3%) required intensive care, and 70 (80.5%) were discharged. Additionally, follow-up blood samples collected on day 9 of hospitalization were available for 40 patients. With regard to treatment, nearly 80% of the patients experienced respiratory dysfunction and required supplemental oxygen therapy. In addition to oxygen administration, patients received antiviral therapy (remdesivir, *n* = 11), immunomodulatory treatment (tocilizumab, *n* = 20; baricitinib, *n* = 3), and corticosteroid therapy (dexamethasone, *n* = 37), which was continued until clinical improvement or death. Remdesivir (Veklury) was administered intravenously at 200 mg on the first day, followed by 100 mg daily on the subsequent 4 days. Tocilizumab (RoActemra) was administered at a dose of 400 mg as a single dose via intravenous infusion to patients with serum IL-6 greater than 100 pg/mL. Baricitinib (Olumiant) was given orally at a dose of 4 mg daily for 10 days to patients with passive oxygen therapy and present risk factors for severe disease. Dexamethasone was administered intravenously at a dose of at least 4 mg per day.

The control group included 45 healthy participants (27 females and 18 males) aged 22–60 years (mean age: 31.7 years). All individuals were asymptomatic, tested negative for SARS-CoV-2 by reverse transcription polymerase chain reaction (RT-PCR), and had no detectable anti-SARS-CoV-2 antibodies. The laboratory characteristics of the study and control groups are summarized in Table 2. Written informed consent was obtained from all participants after providing a detailed explanation of the study. This retrospective study was conducted with the approval of the local research Bioethics Committee of the Medical University of Bialystok (APK.002.497.2021; APK.002.125.2024).

### 2.2. Materials

Blood samples were collected from a peripheral vein at two time points: upon admission, prior to treatment initiation (Sample 1, *n* = 87), and on day 9 of hospitalization (±24 h) (Sample 2, *n* = 40). Samples were allowed to clot fully at room temperature, and serum was separated by centrifugation at 1500× *g* for 5 min and then stored at −80 °C pending analysis. Arterial blood for blood gas analysis and CO-oximetry was collected in standard heparinized tubes. Additional aliquots were collected into EDTA-2 tubes for hematological analysis. For coagulation testing, blood was drawn into tubes containing 3.2% sodium citrate to obtain citrated plasma.

### 2.3. Methods

#### 2.3.1. Determination of Hepcidin

The serum hepcidin concentrations (expected range 79.0–49,400 pg/mL) were quantified using a commercially available quantitative sandwich enzyme-linked immunosorbent assay using the Quantikinine ELISA Human Hepcidin Immunoassay kit (R&D Systems, Minneapolis, MN, USA) in accordance with the manufacturer’s protocol. Absorbance was measured at 450 nm using an Anthos 2020 microplate reader (Instruments GmbH, Wals, Austria), and hepcidin concentrations were determined from a standard calibration curve. Samples with levels exceeding the assay’s linear range (up to 1000 pg/mL) were appropriately diluted and reanalyzed, with final concentrations calculated after applying the corresponding dilution factor. The minimum detectable dose (MDD) of protein ranged from 0.446 to 3.81 pg/mL (the mean MDD was 1.70 pg/mL). The total precision (CV, coefficient of variance) was 4.3% at 175 pg/mL, 3.1% at 350 pg/mL, and 3.2% at 726 pg/mL.

#### 2.3.2. Calculation of Transferrin Saturation (TSAT)

The standard formula for transferrin saturation (TSAT) is:

TS (%) = (Serum iron/TIBC) × 100,

where serum iron represents the concentration of circulating iron and TIBC (total iron-binding capacity) is an indirect measure of transferrin concentration.

The result is expressed as a percentage (%). The typical clinical reference range for adults is 20–50%.

#### 2.3.3. Biochemical Measurements

Biochemical assays, such as serum alanine aminotransferase (ALT; expected range for men is 5–41 IU/L and for women is 5–33 IU/L), aspartate aminotransferase (AST; expected range: 5–36 IU/L), γ-glutamyltransferase (GGT; expected range for men is 10–71 IU/L and for women is 6–42 IU/L), lactate dehydrogenase (LDH; expected range for men is 135–225 IU/L and for women is 135–214 IU/L), creatine kinase (CK; expected range for men is 20–200 IU/L and for women is 20–180 IU/L), total bilirubin (expected values for adults are up to 1.2 mg/dL), glucose (expected range is 70–99 mg/dL), cholesterol (expected values are up to 200 mg/dL), creatinine (expected range for men is 0.70–1.20 mg/dL and for women is 0.50–0.90 mg/dL), iron (expected range for men is 59–158 µg/dL and for women is 37–145 µg/dL), C-reactive protein (CRP; expected values are below 5 mg/L), transferrin (expected range is 2.0–3.6 g/L), soluble transferrin receptor (sTfR; expected range is 1.71–4.13 mg/L), and ferritin (expected range for men is 30–400 µg/L and for women is 15–150 µg/L), were measured using routine laboratory methods on a COBAS c501 analyzer (Roche/Hitachi, Tokyo, Japan). Interleukin 6 (IL-6; expected values are below 7 pg/mL) and procalcitonin (PCT; 95th percentile is below 0.05 ng/mL) were determined by electrochemiluminescence assay on COBAS e-411 (Roche Diagnostics, Rotkreuz, Switzerland). Interleukin 18 (IL-18; expected range: 84.8–358 pg/mL) was measured separately via the quantitative sandwich enzyme immunoassay technique using the Quantikine ELISA Human Total IL-18/IL-1F4 Immunoassay Kit (R&D Systems, Minneapolis, MN, USA). Interferon gamma (INF-γ) and tumor necrosis factor alpha (TNF-α) were determined via the enzyme-linked immunosorbent assay using the Invitrogen Human IFN gamma ELISA Kit and the Invitrogen Human TNF alpha ELISA kit (Thermo Fisher Scientific, Vienna, Austria) using the Anthos 2020 microplate reader set at 450 nm (Instruments GmbH, Wals, Austria).

#### 2.3.4. Hematological Assays

A complete blood count (CBC) was determined on the Sysmex XN-1000 analyzer (Sysmex Corporation, Singapore). The following reference values were used: RBC: 4.2–5.4 × 10^6^/µL in men and 3.5–5.2 × 10^6^/µL in women, Hb: 14–18 g/dL in men and 12–16 g/dL in women, Ht: 40–54% in men and 37–47% in women, MCV: 82–93 fL, WBC: 4–10 × 10^3^/µL, and PLT: 125–400 × 10^3^/µL.

#### 2.3.5. Coagulometric Test

Coagulometric tests, such as prothrombin time (PT; reference range is 12–16 s), international normalized ratio (INR; reference range is 0.8–1.2), and fibrinogen (reference range is 180–400 mg/dL), were performed on an ACL TOP 300 CTS analyzer (Instrumentation Laboratory, Werfen Company, Bedford, MA, USA).

#### 2.3.6. Arterial Blood Gas (ABG) and CO-Oximetry

Arterial blood gas and CO-oximetry were performed on an ABI 90 FLEX PLUS analyzer (Radiometer Medical ApS, Brønshøj, Denmark). The normal arterial blood gas and CO-oximetry were as follows: pH—7.35–7.45, PaO_2_—75–100 mmHg, PaCO_2_—32–45 mmHg, BE—from −2.5 to +2.5, and O_2_Sat—95–98%.

### 2.4. Statistics

Statistical analysis was performed using Statistica 13.3 PL (StatSoft, Cracow, Poland). The results were expressed as medians and interquartile ranges (Q1–Q3). The normality of distribution was checked by means of the Kolmogorov–Smirnov test. The analysis revealed a normal distribution of Hb, RBC, MCV, PLT, and PaO_2_. For the tests with a normal distribution, the differences between Sample 1 and controls and Sample 2 and controls were estimated by Student’s *t*-test, whereas for the tests with a non-normal distribution, the Mann–Whitney U test was used. The differences between Sample 1 and Sample 2 were analyzed by means of the Wilcoxon matched-pairs test in 40 patients with paired samples available at both time points. The effect of the disease severity on the concentration of iron-related parameters was tested using the Kruskal–Wallis test followed by a further post hoc analysis. For tests with a non-normal distribution, Spearman’s rank correlation coefficient was used, while for those with a normal distribution, Pearson’s correlation test was used. The results were considered statistically significant when *p*-values were less than 0.05. For comparative purposes only, the Benjamini–Hochberg false discovery rate (FDR) correction was applied; however, given the exploratory nature of the study, it was not incorporated into the primary analyses.

## 3. Results

The results of basic laboratory tests of COVID-19 patients and controls are presented in Table 2. Upon admission to the hospital, among biochemical findings, the median serum activity of ALT, AST, GGT, LDH, and glucose and cholesterol concentration were significantly elevated compared to the controls (for all comparisons *p* < 0.001), whereas after hospitalization, a significant decrease was observed in AST, LDH, and CK activity (*p* = 0.005, *p* < 0.001, and *p* < 0.001, respectively) and in creatinine levels (*p* = 0.001). All inflammatory markers (IL-6, IL-18, CRP, procalcitonin, and haptoglobin) were significantly increased in patients upon admission compared to the controls (for all comparisons *p* < 0.001), while after hospitalization, a significant decrease was noted in CRP, IL-18, and haptoglobin concentration (for all comparisons *p* < 0.001). Among the hematological parameters, Hb, Ht, and RBC were significantly lowered, and MCV was elevated in COVID-19 patients upon hospital admission compared to healthy subjects (*p* < 0.001, *p* = 0.028, *p* < 0.001, *p* = 0.001, respectively). After hospitalization of these patients, the MCV value and platelet count were increased (*p* = 0.041, *p* = 0.002, respectively), and the white blood cell count was significantly decreased (*p* = 0.047). The values of all coagulation tests (PT, INR, fibrinogen) were increased in patients upon admission compared to the controls (for all comparisons *p* < 0.001) and did not change after hospitalization. Among the blood gas parameters, pH and BE were significantly increased, whereas PaO_2_ and PaCO_2_ were decreased in patients with SARS-CoV-2 infection upon hospital admission compared to the controls (*p* < 0.001, *p* = 0.002, *p* = 0.008, *p* = 0.002, respectively).

**Figure 2:**
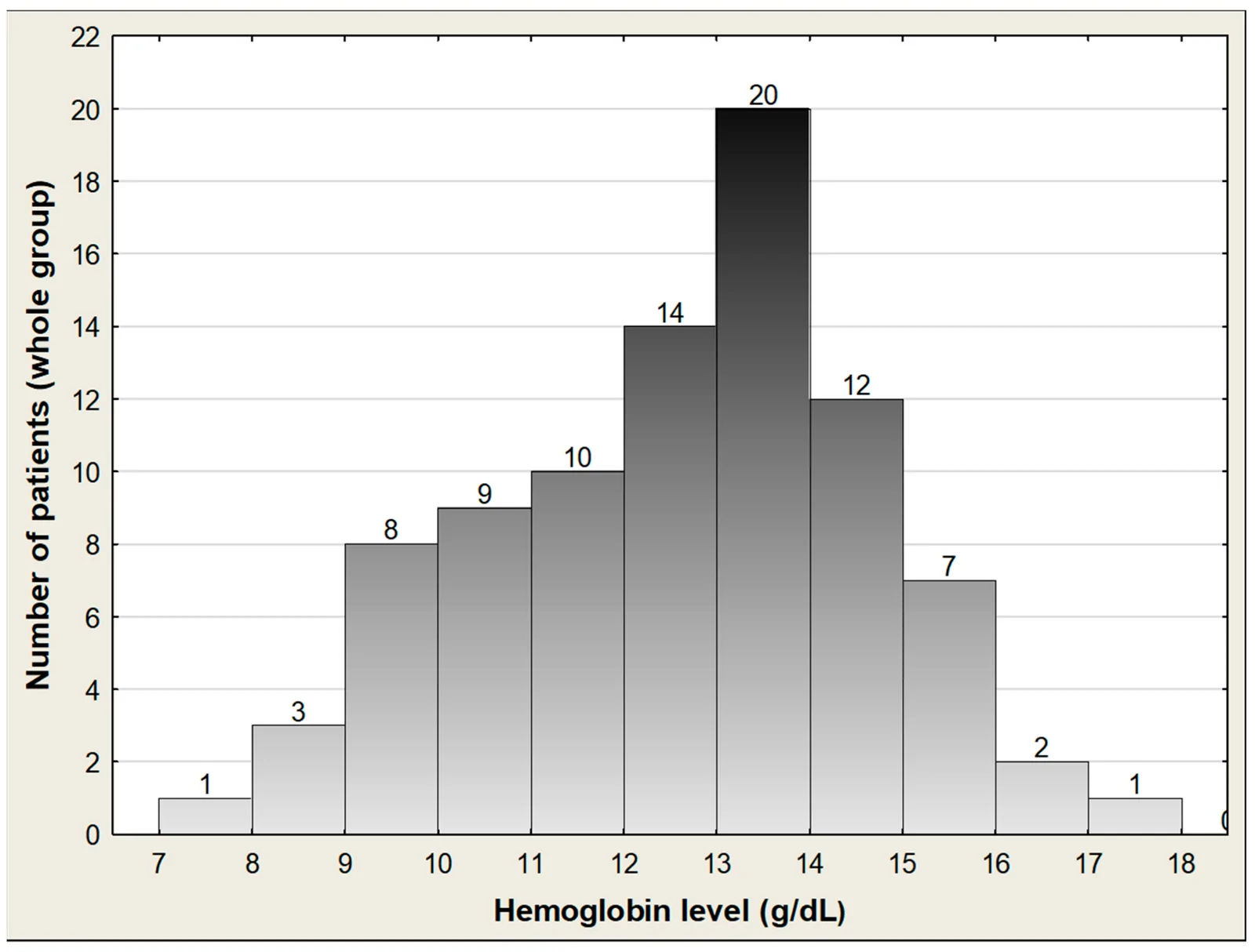
The distribution of hemoglobin concentration in the whole study population.

**Figure 3:**
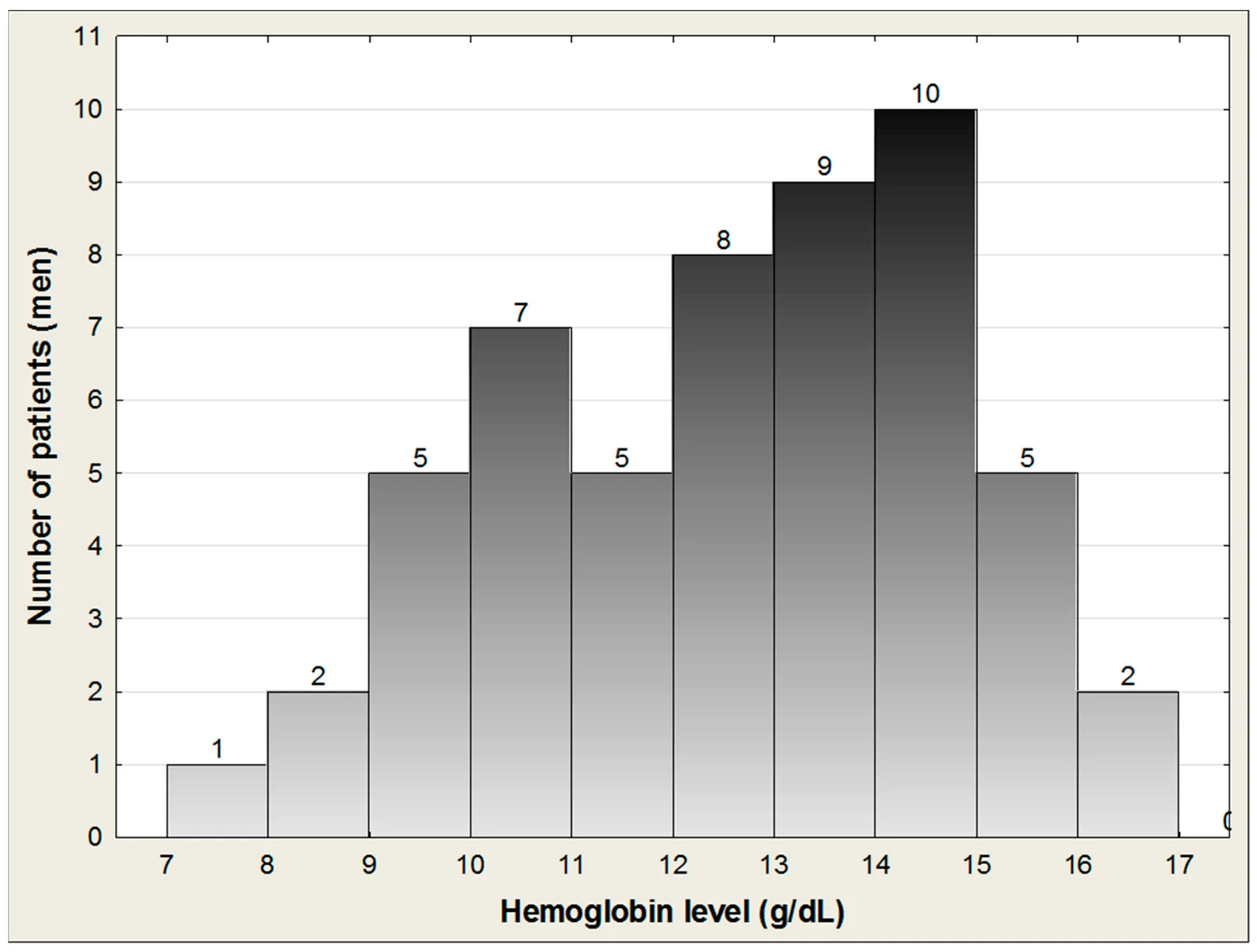
The distribution of hemoglobin concentration in the study population (men).

**Figure 4:**
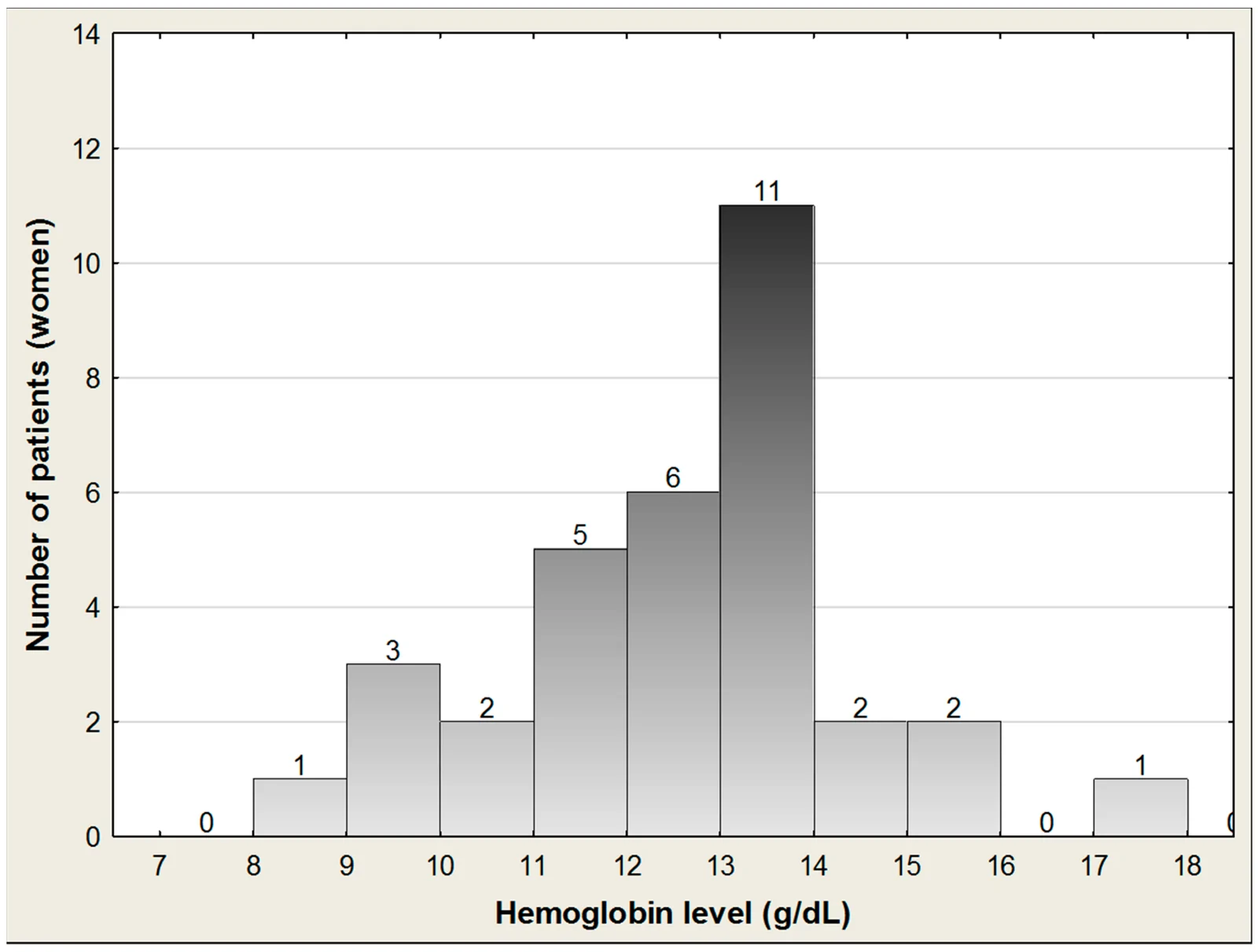
The distribution of hemoglobin concentration in the study population (women).

**Figure 5:**
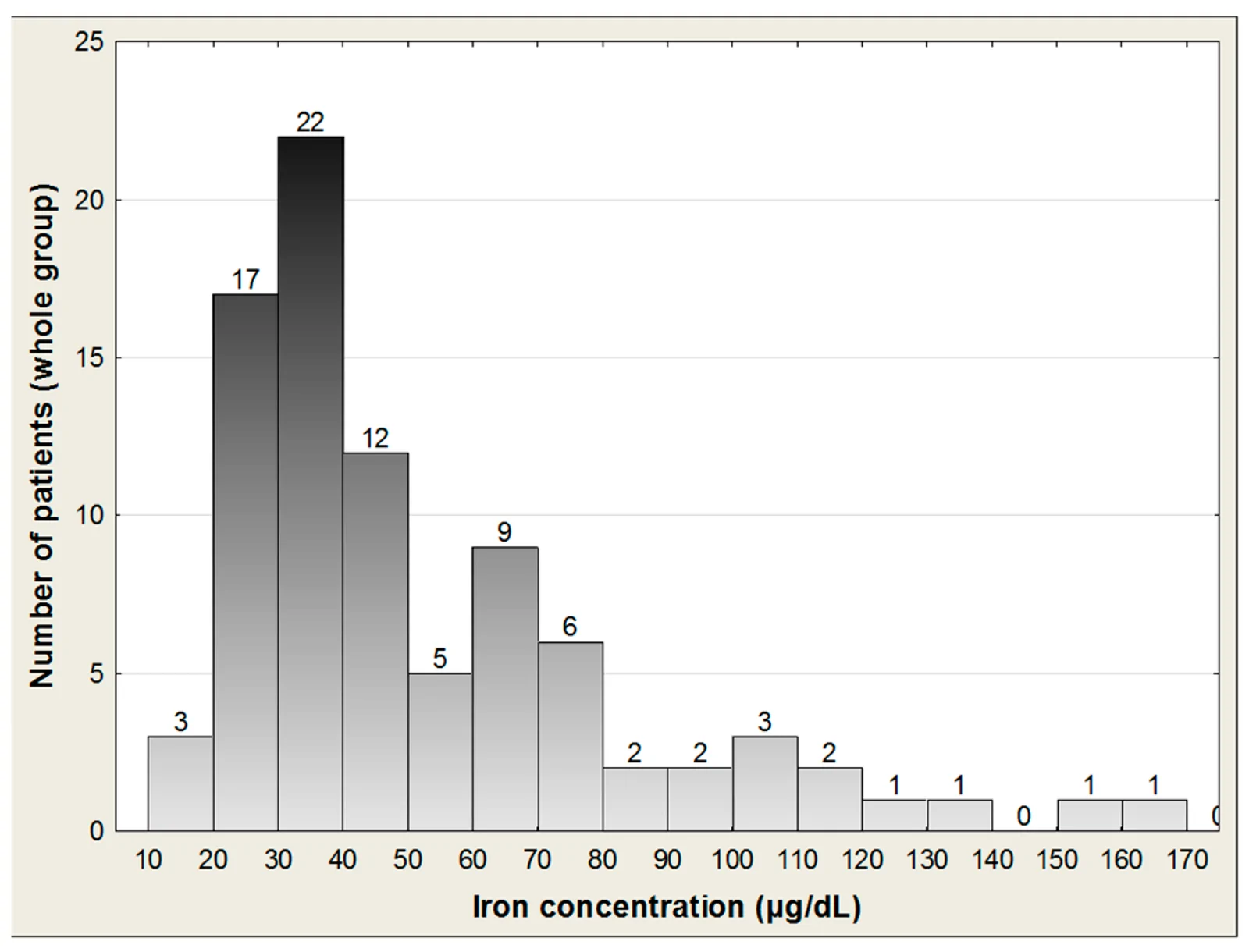
The distribution of iron concentration in the whole study population.

**Figure 6:**
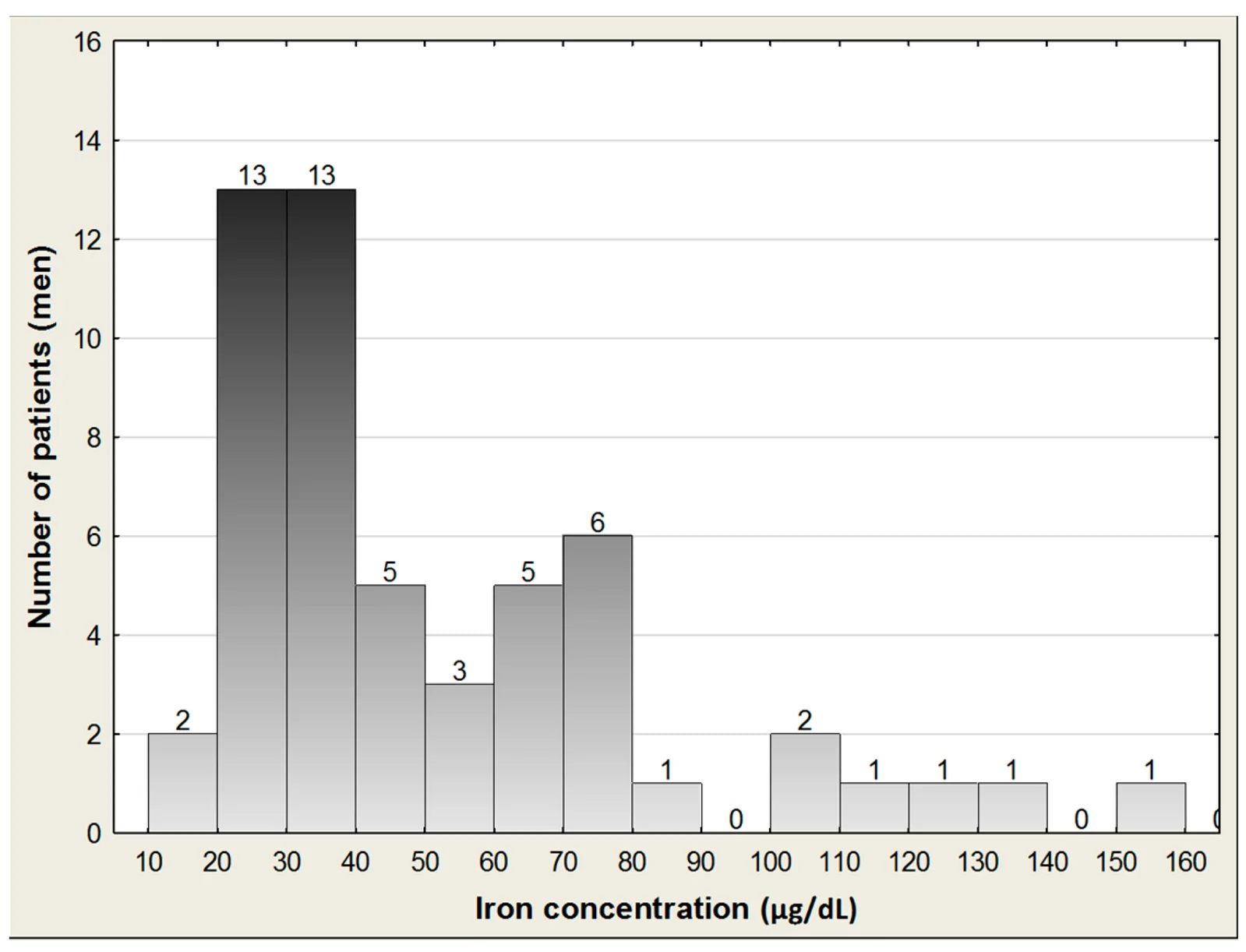
The distribution of iron concentration in the study population (men).

**Figure 7:**
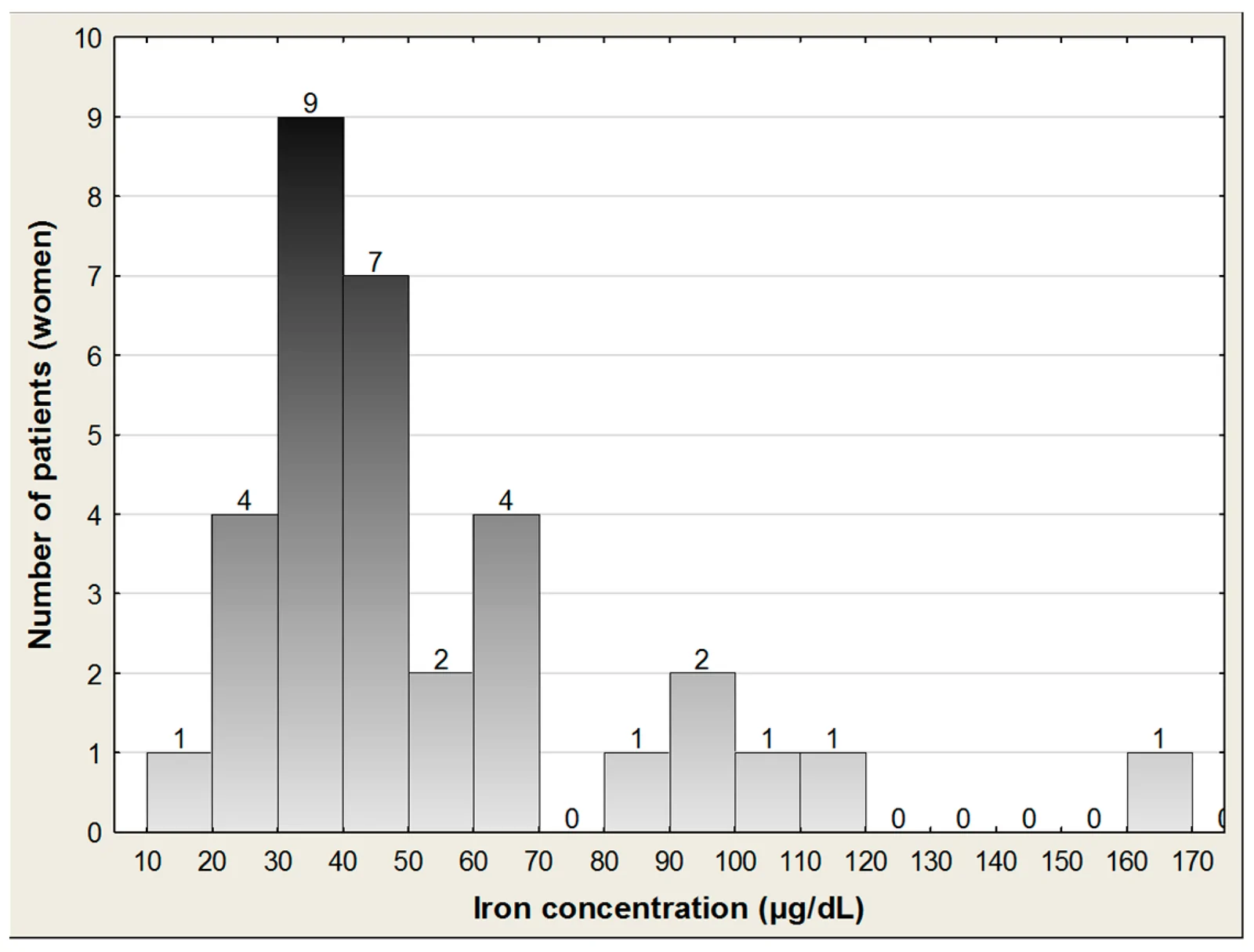
The distribution of iron concentration in the study population (women).

Figure 2, Figure 3 and Figure 4 illustrate the distribution of hemoglobin concentrations in the entire COVID-19 cohort and by sex. Among the 33 women included in the study, 11 (33.3%) had Hb levels below the reference range (<12–16 g/dL), whereas reduced Hb concentrations (<14–18 g/dL) were observed in 37 out of the 54 men (68.5%). Figure 5, Figure 6 and Figure 7 present the distribution of iron concentrations in the entire COVID-19 group and stratified by sex. Reduced iron levels predominated among male patients, with 36 cases (66.7%) below the reference limit of 59–158 µg/dL. The proportion was lower among female patients, with 14 cases (42.4%) below 37–145 µg/dL.

**Figure 8:**
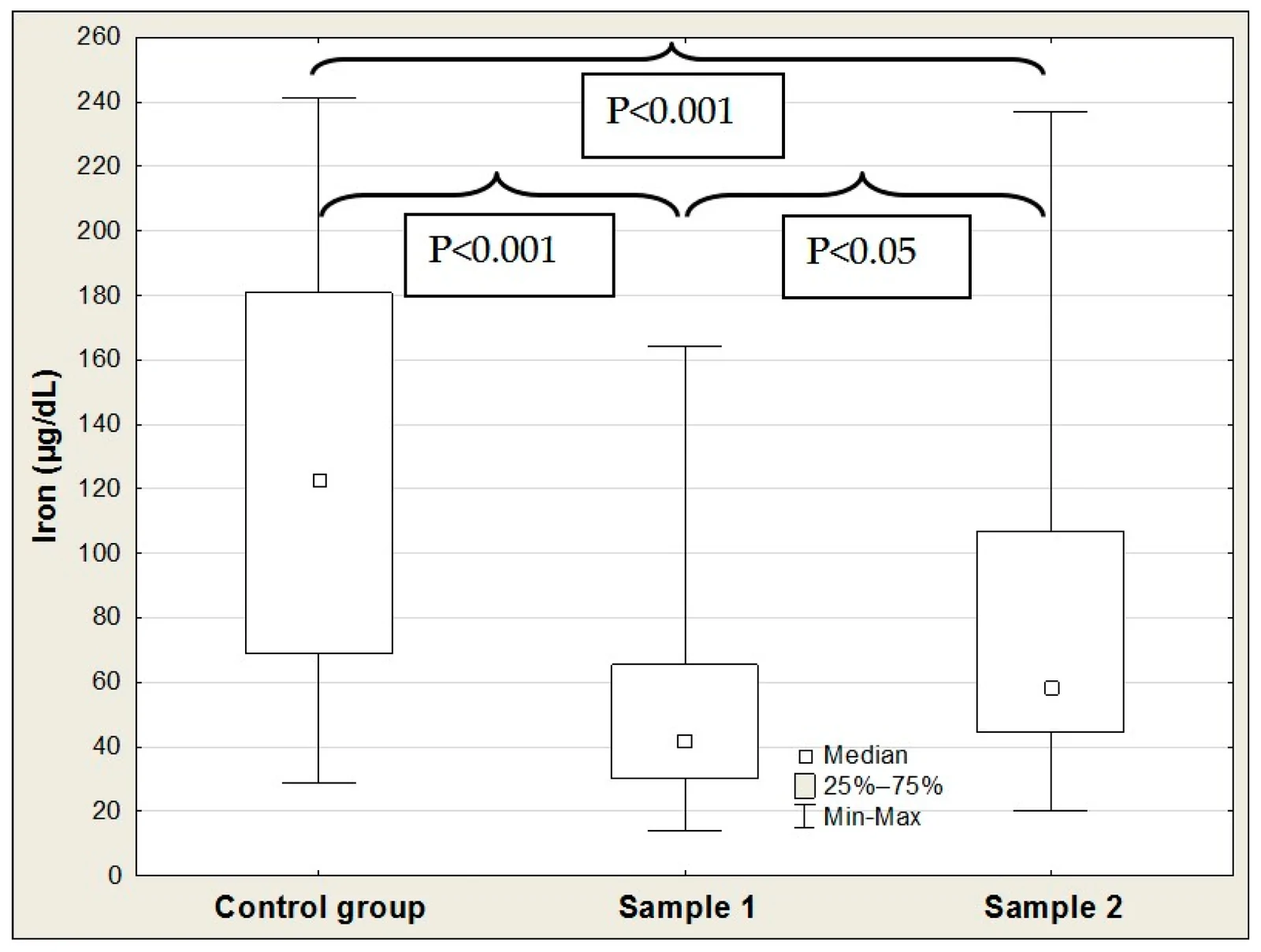
Iron concentration in the control group and COVID-19 patients on admission (Sample 1) and after hospitalization (Sample 2).

**Figure 9:**
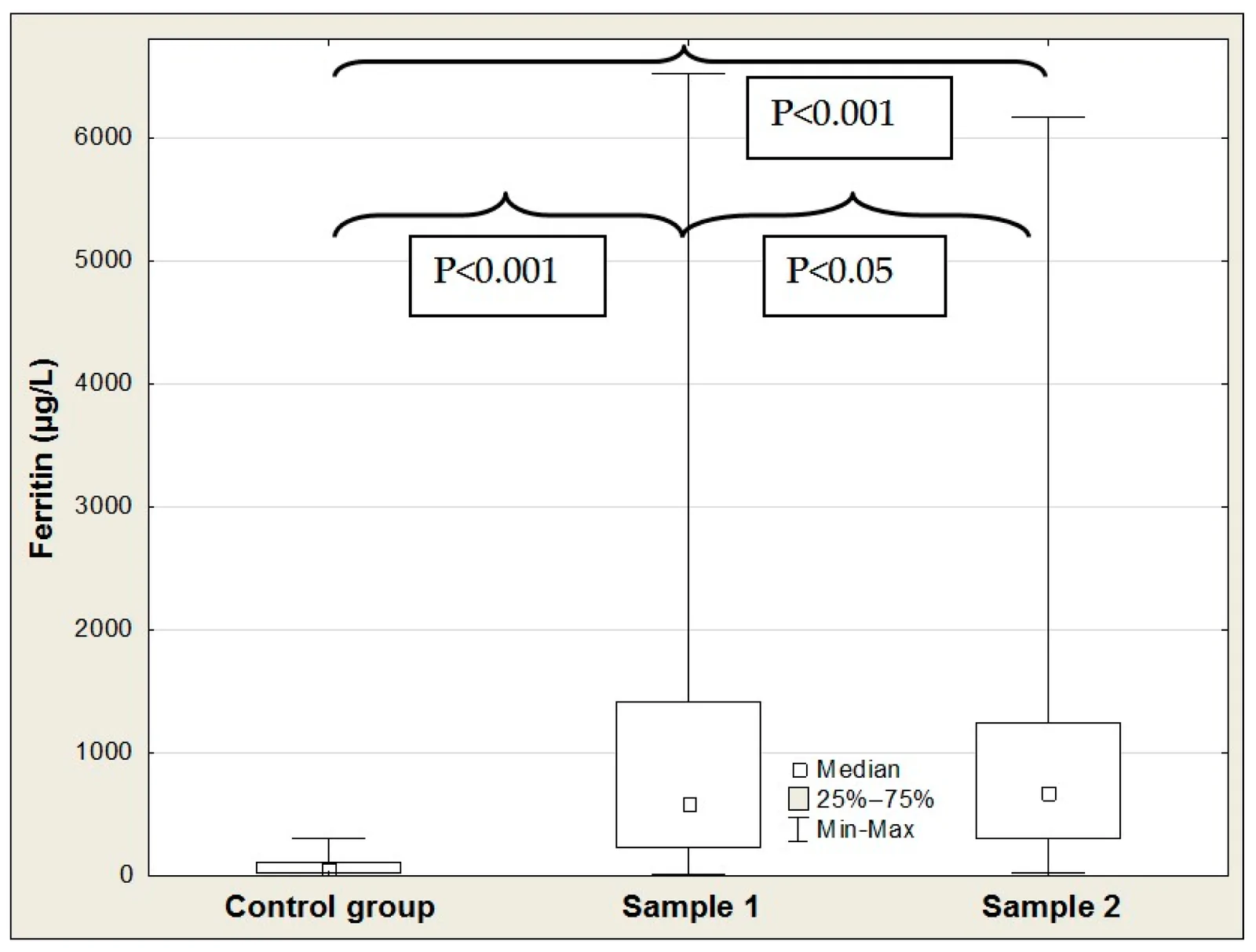
Ferritin concentration in the control group and COVID-19 patients on admission (Sample 1) and after hospitalization (Sample 2).

**Figure 10:**
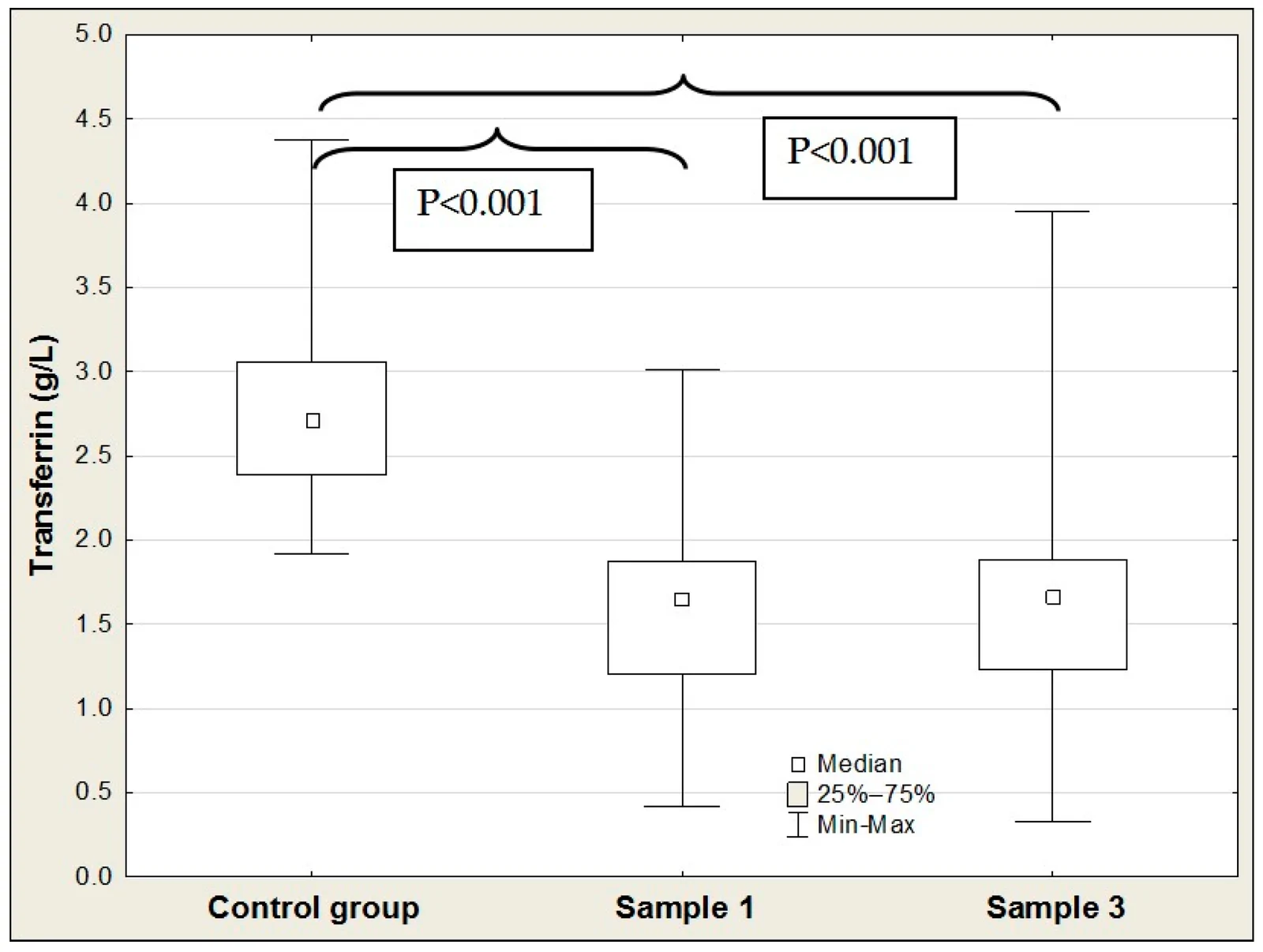
Transferrin concentration in the control group and COVID-19 patients on admission (Sample 1) and after hospitalization (Sample 2).

**Figure 11:**
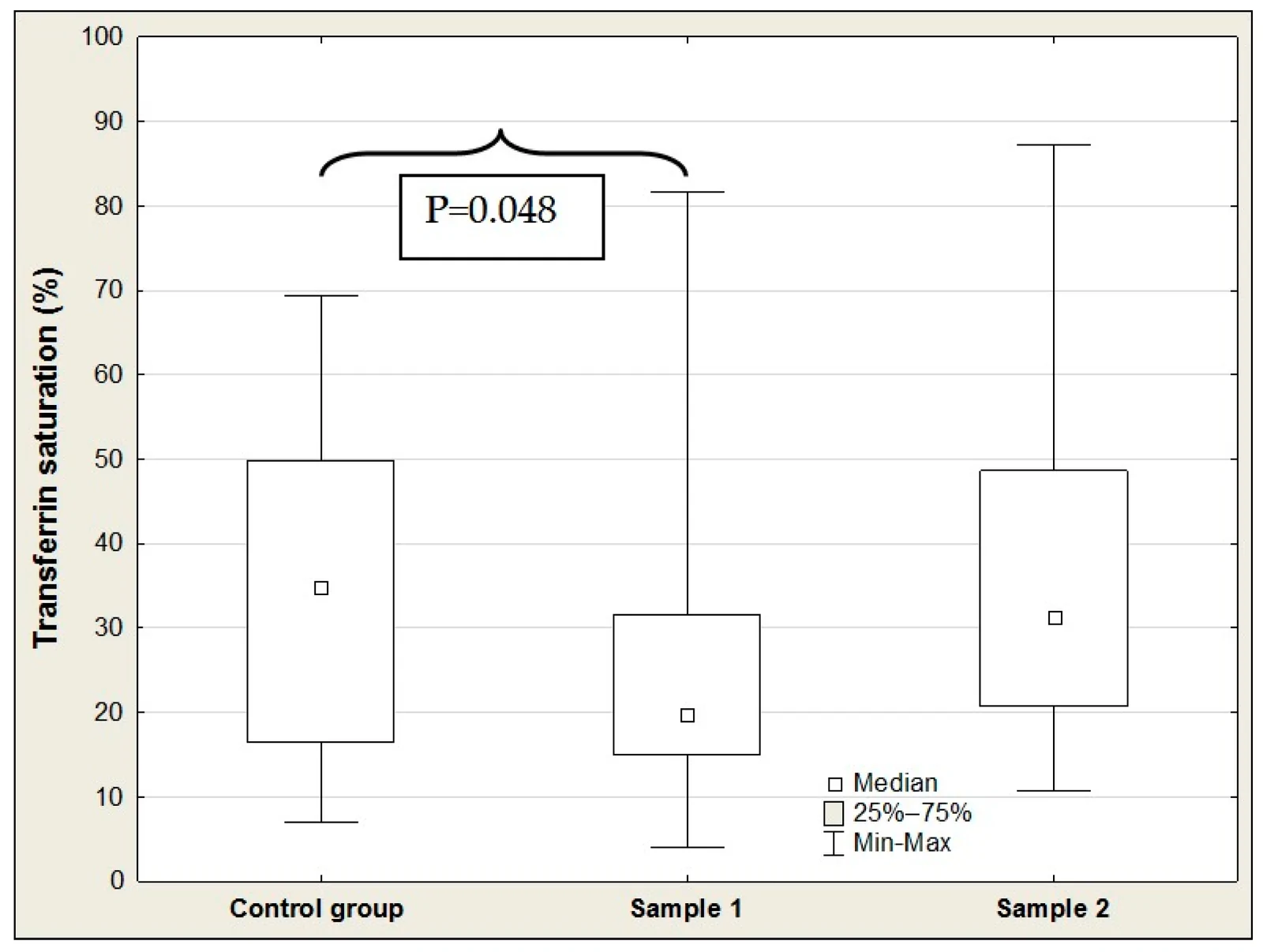
Transferrin saturation in the control group and COVID-19 patients on admission (Sample 1) and after hospitalization (Sample 2).

**Figure 12:**
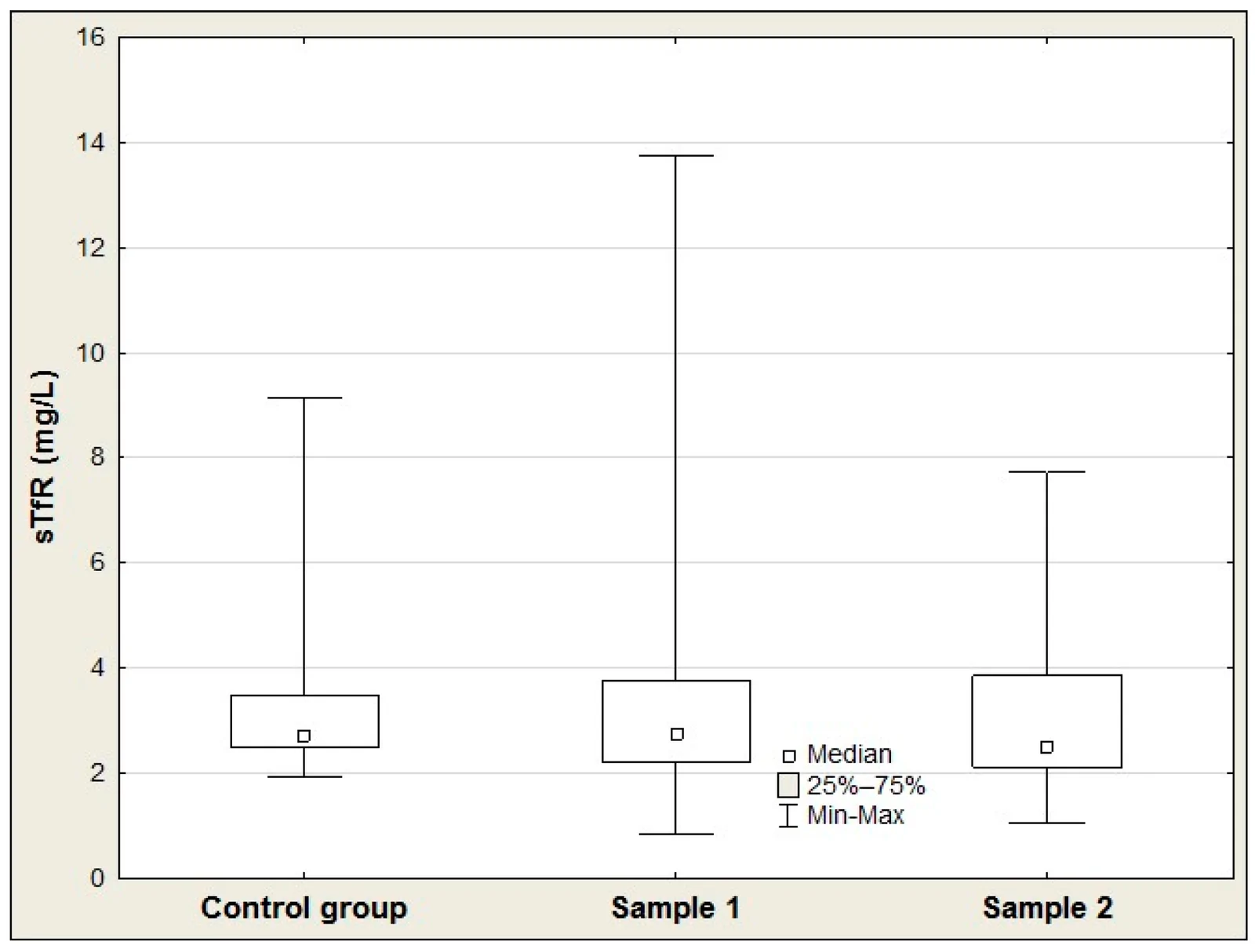
Soluble transferrin receptor in the control group and COVID-19 patients on admission (Sample 1) and after hospitalization (Sample 2).

**Figure 13:**
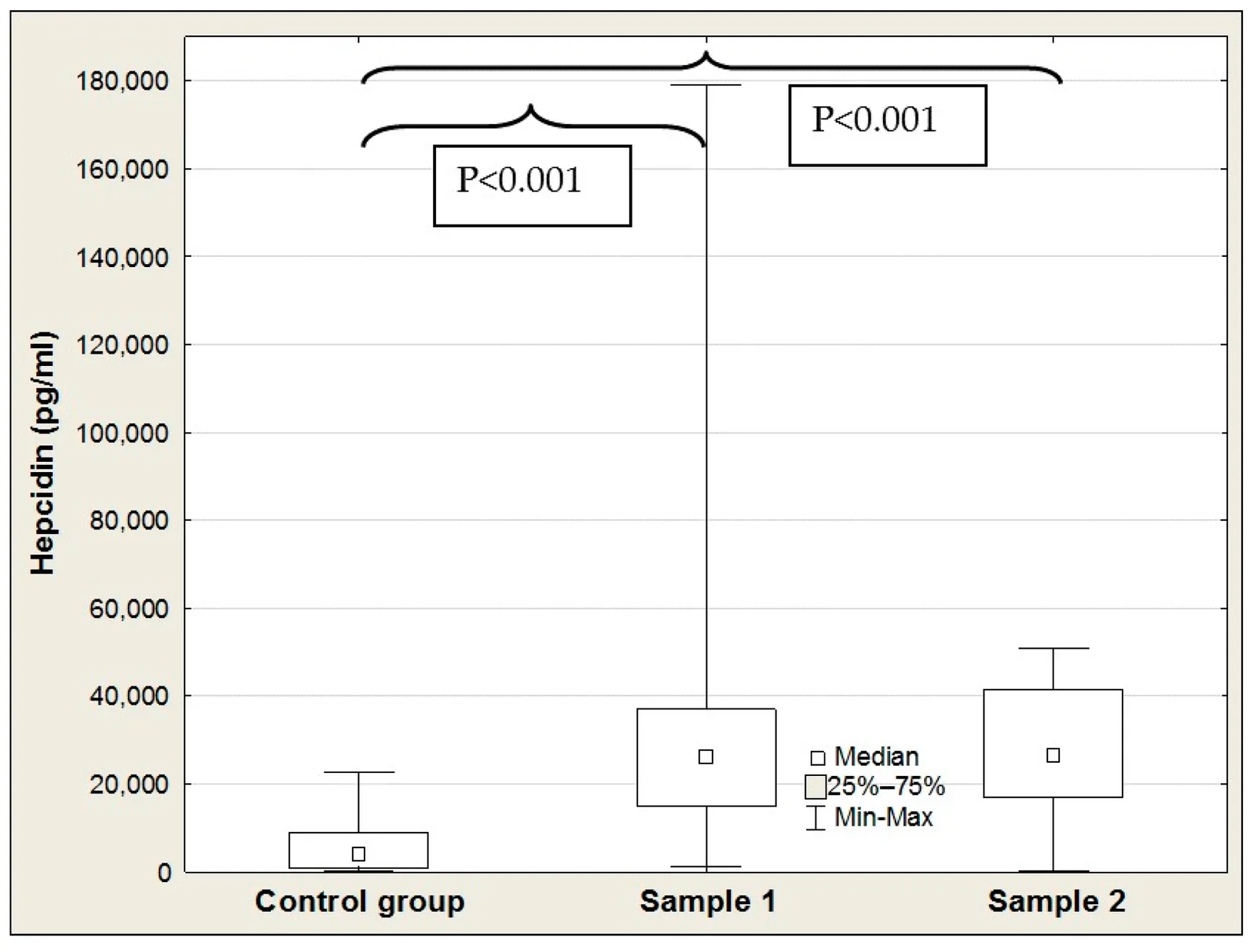
Hepcidin concentration in the control group and COVID-19 patients on admission (Sample 1) and after hospitalization (Sample 2).

Table 3 shows the results of iron status markers in COVID-19 patients and controls. The median serum iron, transferrin concentrations and transferrin saturation were significantly decreased (42.1 vs. 128.8 µg/dL, *p* < 0.001; 1.66 vs. 2.72 g/L, *p* < 0.001; 19.8 vs. 35.0 (%), *p* = 0.048, respectively) in the COVID-19 patients on admission (Sample 1), whereas the ferritin and hepcidin concentrations were increased (593 vs. 54.5, *p* < 0.001; 26,413 vs. 4194 pg/mL, *p* < 0.001) in comparison to the control group. In turn, the serum iron and ferritin levels were significantly increased (58.6 vs. 42.1 µg/dL, *p* < 0.05; 672 vs. 593 µg/L, *p* < 0.05) (Figure 8 and Figure 9) after hospitalization (Sample 2) compared to those before admission to the hospital. The median serum sTfR concentrations remained unchanged across all of these comparisons. The results are graphically presented in Figure 8, Figure 9, Figure 10, Figure 11, Figure 12 and Figure 13. 

Taking into account the gender of patients, among all iron markers, only median ferritin concentration was shown to be significantly higher in men compared to the values in women (682 vs. 367 µg/L, *p* = 0.011) (Table 4).

Table 5 presents the results of iron markers in the different patient groups according to the cytokine storm, comorbidities, vaccination, and those surviving/non-surviving. The serum ferritin level and transferrin saturation were higher, whereas the transferrin concentration was decreased in patients with cytokine storm compared to those without it (1367 vs. 391 µg/L, *p* < 0.001; 22.9 vs. 18.8%, *p* = 0.011; 1.20 vs. 1.77 g/L, *p* < 0.001, respectively). There were no statistically significant differences in the levels of iron markers between patients with concomitant chronic diseases and patients with and without chronic comorbidities. Analyses of patients’ survival demonstrated that, among all iron-related biomarkers, ferritin concentrations were significantly decreased, whereas transferrin levels were significantly increased in the surviving patients compared to those who did not survive (1271 vs. 523 µg/L, *p* = 0.015; 1.15 vs. 1.75 g/L, *p* = 0.001, respectively). Similar to transferrin, serum iron levels were higher in survivors than in non-survivors; however, this difference only approached statistical significance (34.2 vs. 47.0 µg/dL, *p* = 0.058).

**Figure 14:**
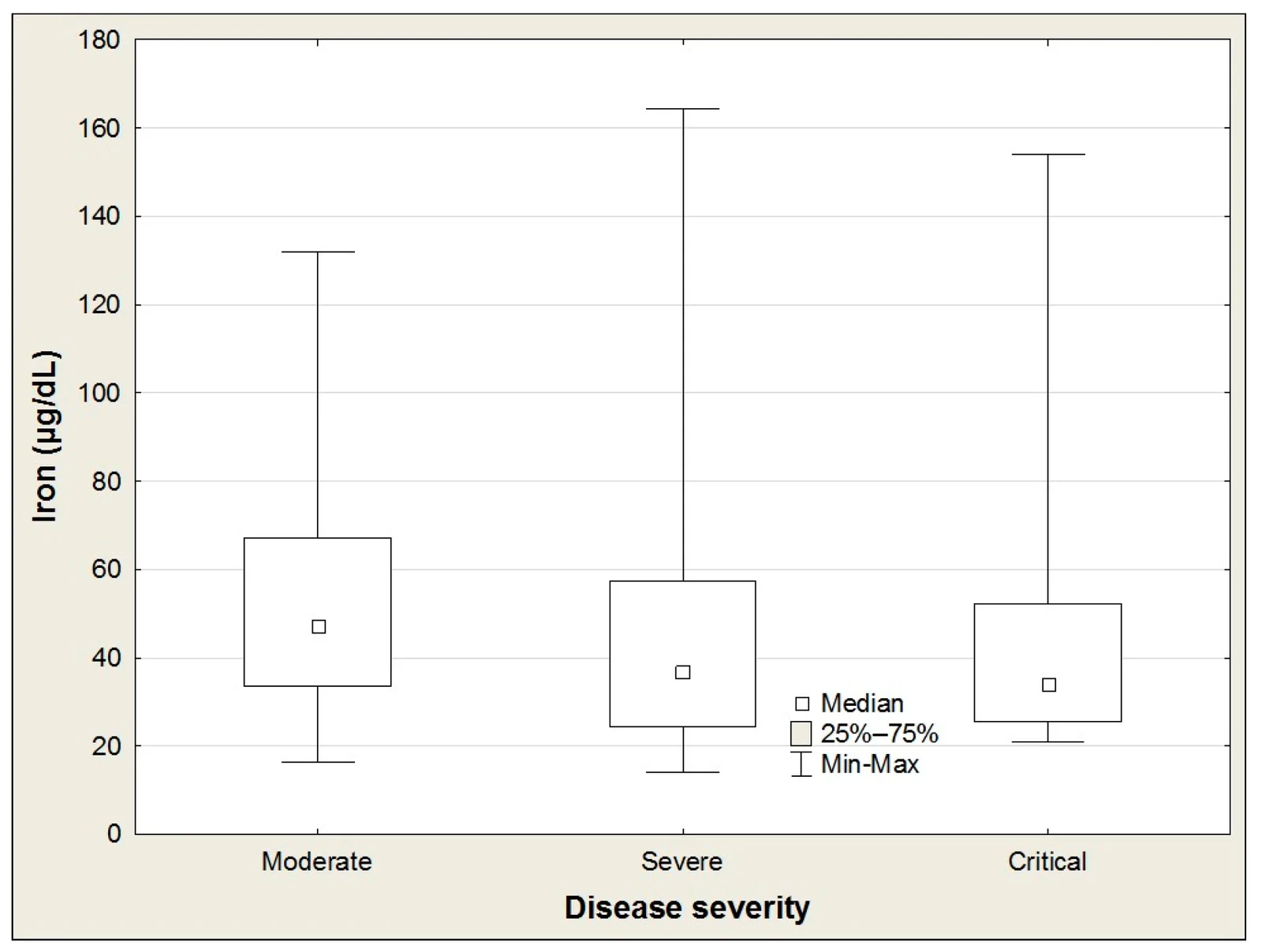
Iron concentration in the COVID-19 patients according to disease severity.

**Figure 15:**
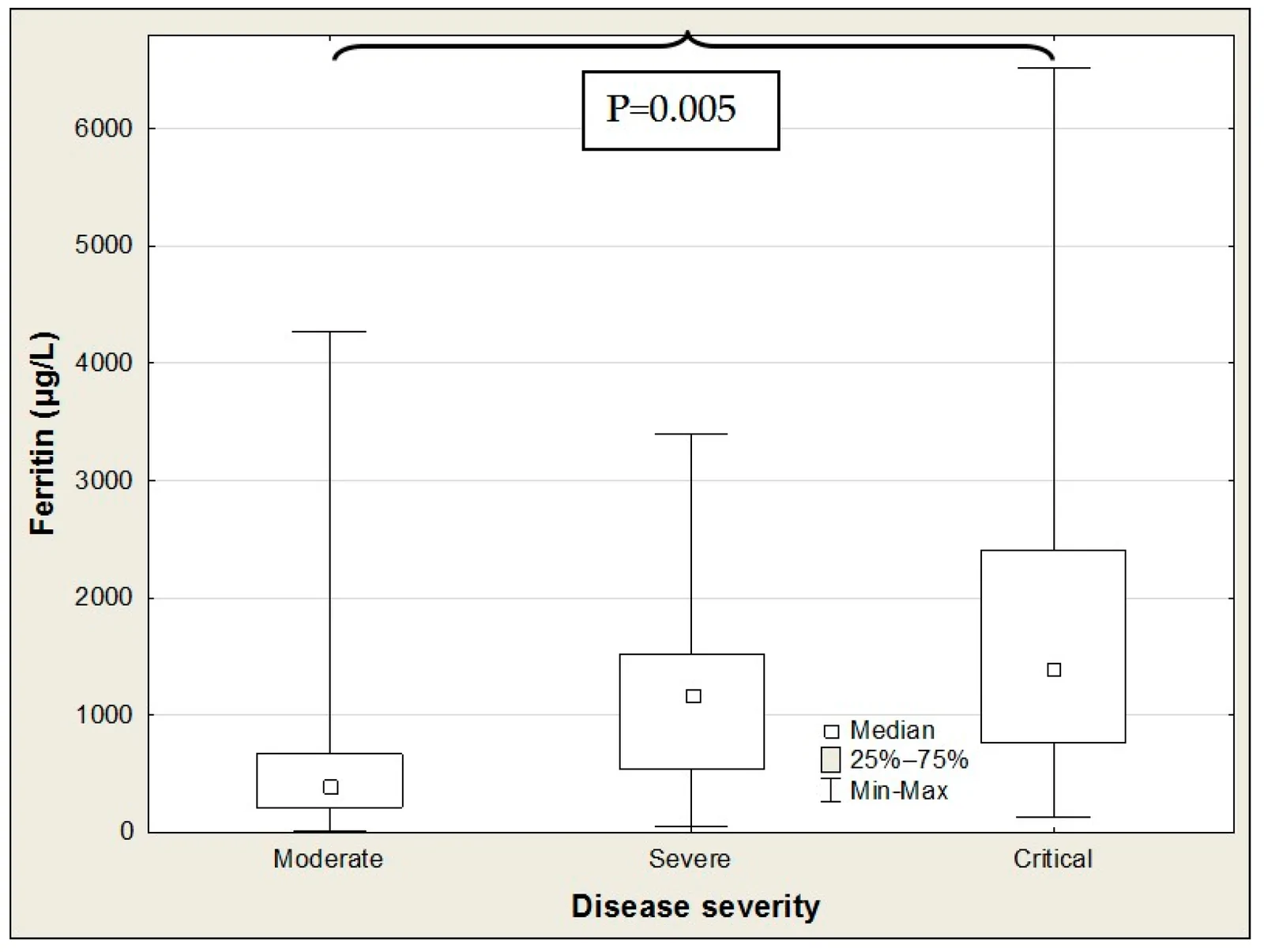
Ferritin concentration in the COVID-19 patients according to disease severity.

**Figure 16:**
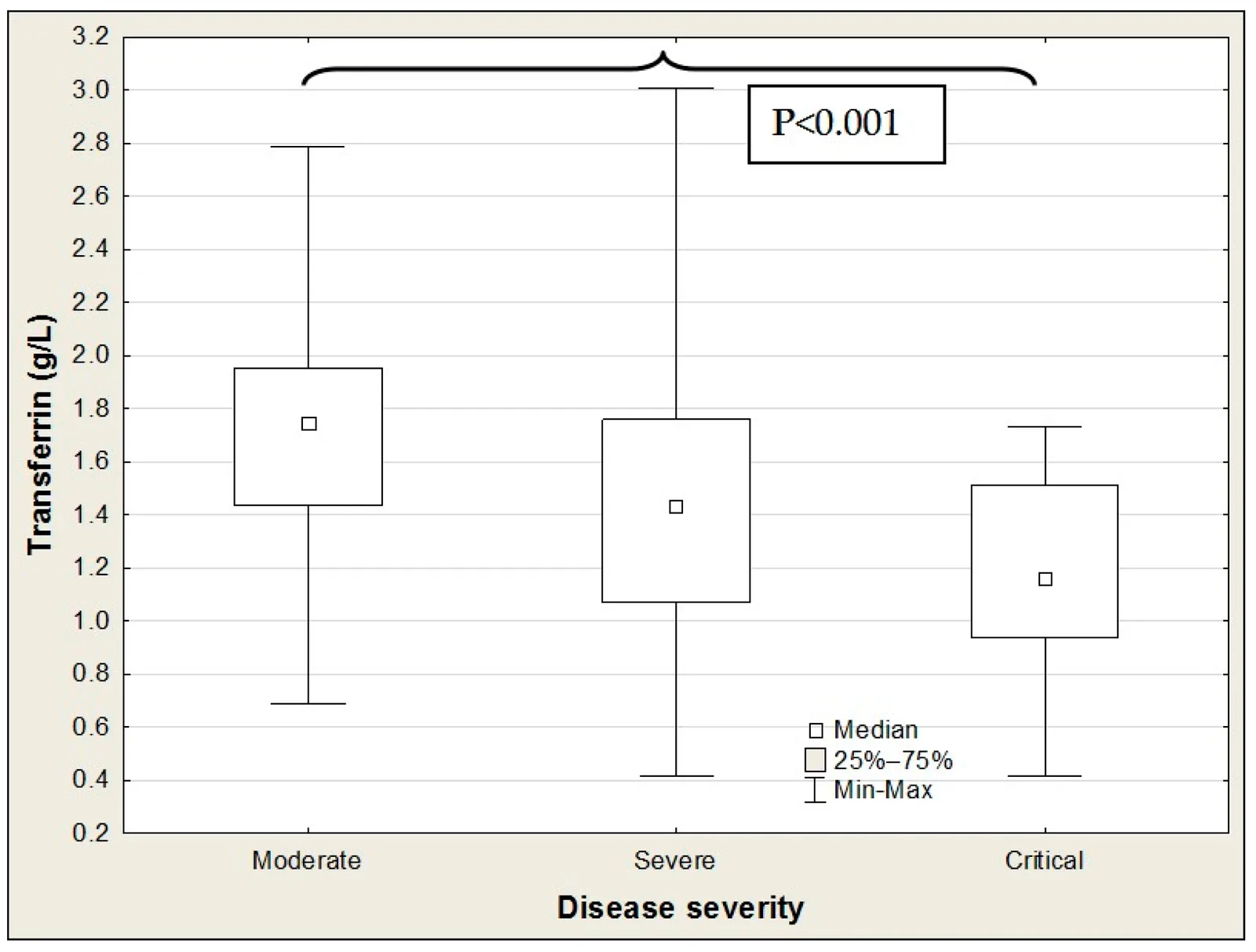
Transferrin concentration in the COVID-19 patients according to disease severity.

**Figure 17:**
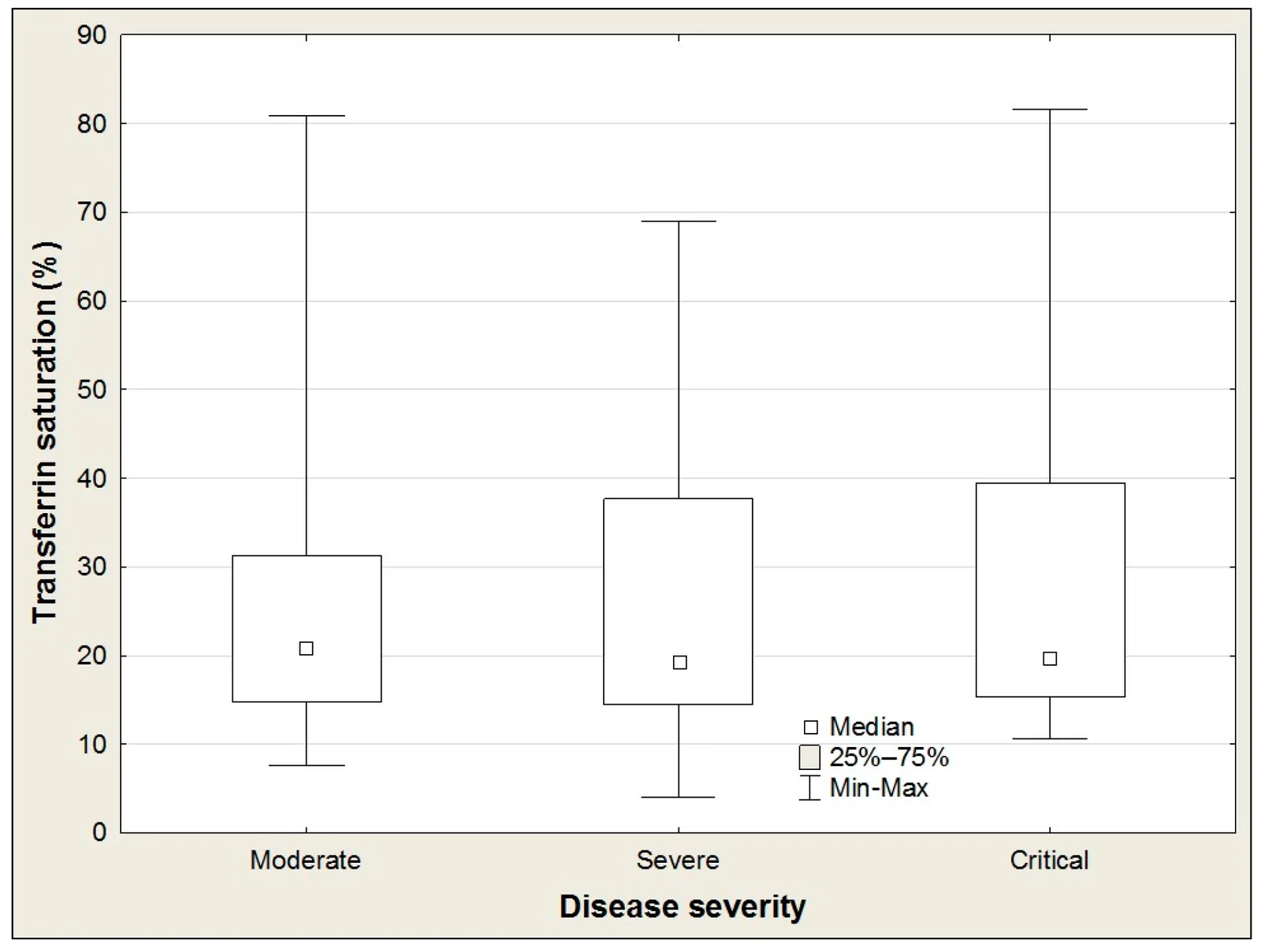
Transferrin saturation in the COVID-19 patients according to disease severity.

**Figure 18:**
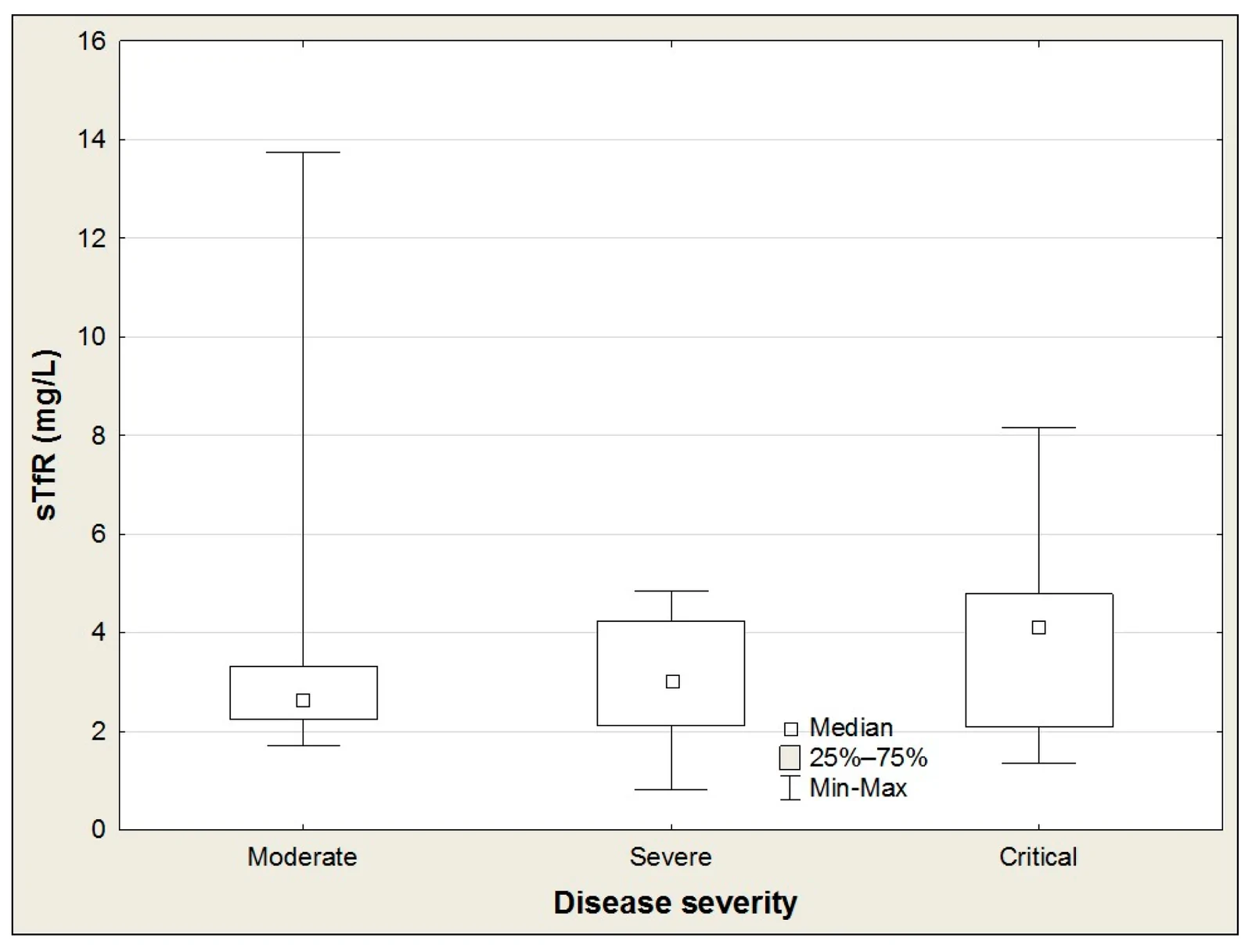
Soluble transferrin receptor concentration in the COVID-19 patients according to disease severity.

**Figure 19:**
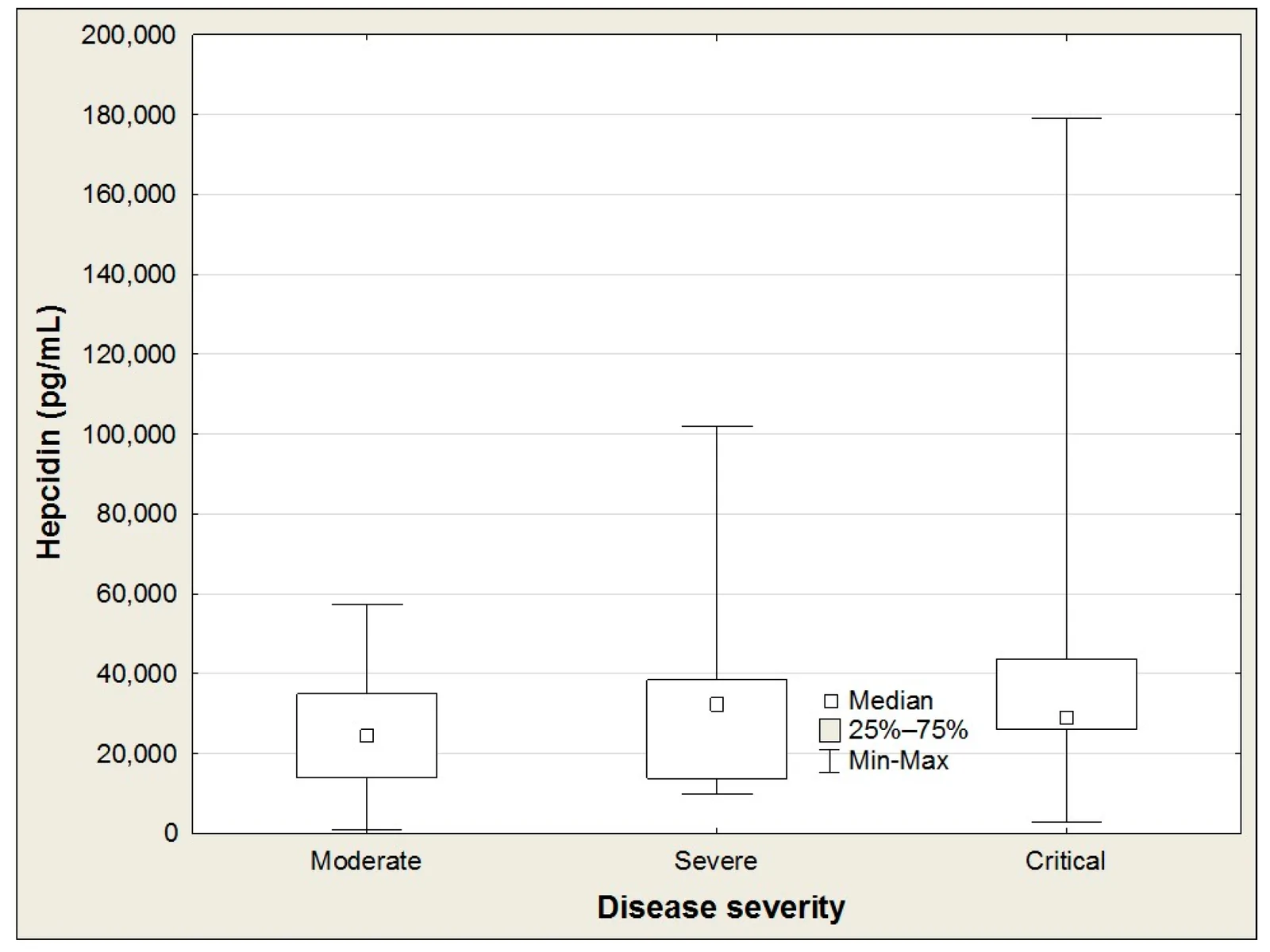
Hepcidin concentration in the COVID-19 patients according to disease severity.

Table 6 summarizes the levels of iron-related markers stratified by disease severity. Thus, serum iron concentrations demonstrate a declining trend with increasing disease severity (Figure 14). Conversely, ferritin levels increased with disease severity, showing a significant elevation in critically ill patients compared to those with moderate disease, while the difference between severe and moderate cases did not reach statistical significance (1394 vs. 391 µg/L, *p* = 0.005; 1176 vs. 391 µg/L, *p* = 0.081, respectively) (Figure 15). Similar to iron, serum transferrin concentrations also exhibit a decreasing trend with disease progression; however, in critically ill patients, they are significantly lower than in those with moderate disease (1.16 vs. 1.75 g/L, *p* < 0.001) (Figure 16). Furthermore, transferrin saturation did not depend on disease severity (Figure 17). The subsequent iron metabolism biomarker, soluble transferrin receptor (sTfR), demonstrates only a rising trend with progressive disease severity (Figure 18). Hepcidin levels in serum exhibit a rising trend, with increased concentrations observed in patients with severe and critical illness relative to those with moderate disease (Figure 19).

Table 7, Table 8, Table 9 and Table 10 show the association between iron markers and selected clinical characteristics and laboratory parameters. With respect to clinical characteristics, transferrin was negatively associated with disease severity (R = −0.210, *p* = 0.040) but showed a negative correlation with cytokine storm (R = −0.473, *p* < 0.001), whereas transferrin saturation and ferritin were positively associated with cytokine storm (R = 0.264, *p* = 0.011; R = 0.354, *p* < 0.001, respectively). None of the iron-related parameters correlated with comorbidities or vaccination status. The correlation between ferritin and patient age approached statistical significance (R = 0.198, *p* = 0.053). The relationships between iron parameters and laboratory findings were as follows: serum iron showed a positive correlation with total bilirubin and a negative association with WBC. Changes in ferritin concentration were significantly positively associated with alterations in the activity of all enzymes, levels of substrates (except cholesterol), inflammatory markers, Hb, and fibrinogen. Transferrin was negatively correlated with selected enzymes (AST, LDH), positively with cholesterol, negatively with inflammatory indices and hematological parameters (positively: Hb, Ht, RBC; negatively: MCV, WBC), and negatively with coagulation tests (Table 7 and Table 8). Furthermore, transferrin saturation showed positive correlation with LDH, selected inflammatory markers (IL-6 and IL-18), and MCV. Soluble transferrin receptor (sTfR) was significantly associated with GGT, total bilirubin, glucose, and coagulation parameters, and negatively correlated with partial oxygen pressure (PaO_2_) and hemoglobin oxygen saturation (O_2_Sat). Changes in hepcidin levels were associated with alterations in GGT activity, concentrations of some substrates (total bilirubin, glucose, and creatinine), inflammatory markers (except IL-6 and procalcitonin), and selected hematological parameters (Hb, Ht, RBC) (Table 9 and Table 10). Following the Benjamini–Hochberg false discovery rate (FDR) correction, the correlations that remained statistically significant were as follows: ferritin with cytokine storm, ALT, AST, GGT, total bilirubin, creatinine, IL-6, IL-18, CRP, procalcitonin, TNF-α, IFN-γ, and fibrinogen; transferrin with cytokine storm, AST, LDH, cholesterol, IL-6, IL-18, procalcitonin, RBC count, and WBC count; transferrin saturation with IL-6 and MCV; sTfR with total bilirubin; and hepcidin with hemoglobin and hematocrit. The above correction was performed for comparative purposes only and, given the exploratory nature of the study, was not incorporated into the primary analyses.

## 4. Discussion

The aim of this study was to assess iron metabolism in patients with SARS-CoV-2 infection requiring hospitalization. Disturbances in iron homeostasis may have serious clinical consequences. On the one hand, iron deficiency can lead to the development of anemia, whereas iron overload may induce oxidative stress, causing cellular injury and contributing to the pathogenesis of various conditions. Although many studies have investigated this issue, the relationship between iron metabolism and COVID-19 remains poorly understood. In our study, iron metabolism was assessed based on alterations in the serum levels of six iron laboratory tests, including iron, ferritin, transferrin, transferrin saturation, soluble transferrin receptor, and hepcidin. Changes in iron parameters may also have diagnostic value in patients with SARS-CoV-2 infection. These biomarkers could be used for the diagnosis and monitoring of COVID-19 and for assessing disease severity and clinical progression. The present study provides a comprehensive evaluation of iron metabolism disturbances in patients with SARS-CoV-2 infection and their relationships with clinical, biochemical, inflammatory, and hematological parameters. The analysis, based on two time points, admission and day 9 of hospitalization (±24 h), allows assessment of both baseline alterations and their evolution during the course of the disease.

The study group was relatively heterogeneous in terms of demographic and clinical characteristics and consisted of 87 patients, with a predominance of males, spanning a wide age range from 22 to 89 years. The number of patients available for sampling on day 9 was lower (40 cases) due to patient death, earlier discharge, or lack of consent for a second blood collection. All individuals were newly diagnosed with SARS-CoV-2 infection and had not received any treatment before hospital admission. The duration of symptoms ranged from 1 day to 3 weeks. Three-quarters of the patients had chronic comorbidities, most commonly hypertension, type 2 diabetes, and liver cirrhosis. None of the patients reported iron-deficiency anemia or anemia of chronic disease (ACD). One-third of the patients were vaccinated, and a similar proportion developed a cytokine storm. Based on disease severity, patients were classified into those with a moderate course (over half of the cohort) and those with severe and critical disease, who together accounted for approximately half the number of patients in the moderate group.

The first parameter evaluated was serum circulating iron. On admission, the median serum iron concentration among patients was significantly lower than that observed in the healthy control group. Although reduced iron levels were common, not all patients exhibited iron deficiency according to the reference range, as a proportion of individuals had serum iron concentrations within the normal range. Sex-specific analysis indicated that decreased iron levels were more prevalent among men than women. It should be emphasized that under physiological conditions, iron levels are typically lower in women than in men. Interestingly, although physiological sex-related differences in iron levels are well documented, with lower baseline values typically observed in women, our findings suggest that this disparity diminishes in the context of SARS-CoV-2 infection. Our findings are consistent with the data reported in the literature. Low serum iron levels have been consistently reported in COVID-19 outpatients and inpatients [6,32]. Evidence from the literature indicates that serum iron concentrations were reduced below normal values in nearly 90% of hospitalized patients with COVID-19 [21,33]. Notably, persistent iron deficiency was observed in 30% of patients even two months after the onset of infection [32]. The observed decrease in serum iron levels in patients with COVID-19 is consistent with the well-established phenomenon of inflammation-driven iron redistribution. In the course of SARS-CoV-2 infection, proinflammatory cytokines, particularly interleukin-6, stimulate hepatic hepcidin production, which in turn inhibits ferroportin-mediated iron export from enterocytes and macrophages [34,35]. As a result, circulating iron levels decline despite relatively preserved or even increased total body iron stores. This mechanism, commonly referred to as hypoferremia of inflammation, serves as a host defense strategy aimed at limiting iron availability for viral replication and secondary infections. The sequestration of iron into ferritin stores limits its availability for erythropoiesis. It is worth noting that approximately 70% of the body’s iron (about 2–3 g) is bound to hemoglobin in red blood cells (RBCs) [36]. Such alterations may exacerbate hypoxia by limiting Hb synthesis and may contribute to fatigue, reduced exercise tolerance, and delayed recovery during and after acute infection [6]. Anemia (as characterized by reduced hemoglobin concentrations) is a common finding among hospitalized patients with COVID-19 and is typically multifactorial [37,38]. While anemia of inflammation predominates, iron-deficiency anemia and mixed etiologies are also observed, particularly in patients with pre-existing comorbidities or poor nutritional status [37]. The hemoglobin concentration in the serum of COVID-19 patients may be normal, reduced, or remain at the lower limit of the reference range [21]. As reported in the literature, decreased Hb levels may be observed in approximately 20% of patients (values below 11 g/dL) and in as many as 50% of affected individuals [8,39,40,41]. In our research, Hb concentrations were below the reference range (<12 g/dL) in 11 out of 33 women included in the study (33.3%), as compared to 37 men showing reduced Hb levels (<14 g/dL) out of all 54 male study patients (68.5%). However, none of the patients fulfilled the diagnostic criteria for iron-deficiency anemia (IDA) or anemia of chronic disease (ACD). It is well established that the diagnosis of IDA requires the presence of anemia accompanied by biochemical evidence of iron deficiency, including decreased serum ferritin levels, reduced transferrin saturation (TSAT < 20%), low serum iron concentration, and elevated transferrin and/or soluble transferrin receptor (sTfR) levels. In contrast, our study demonstrated increased ferritin concentrations, decreased transferrin levels, and no significant changes in sTfR concentrations, findings that are not consistent with the typical laboratory profile of IDA. In turn, ACD is typically characterized by reduced hemoglobin concentrations in the presence of inflammation, low serum iron and transferrin saturation, decreased or normal transferrin levels, and normal or elevated ferritin concentrations. The laboratory pattern observed in our study, namely decreased serum iron and transferrin concentrations together with elevated ferritin levels, is consistent with inflammation-associated anemia. Nevertheless, it should be emphasized that the findings of the present study are consistent with an inflammatory response associated with the acute phase of SARS-CoV-2 infection rather than chronic inflammation. Patients admitted to the hospital with SARS-CoV-2 infection may present with pre-existing anemia, which can be considered a comorbidity, or, more commonly, with anemia resulting from an increased inflammatory response (anemia of inflammation). Some authors have reported that approximately 25% of patients are anemic upon hospital admission, with most cases representing anemia of inflammation [20]. The observed findings may reflect pathophysiological changes consistent with anemia of inflammation and suggest that this mechanism could contribute to the anemia observed in the COVID-19 cohort studied. With the progression of COVID-19, serum iron concentrations showed a decreasing trend. The lack of association suggests that iron depletion may represent a generalized response to infection rather than a marker of disease severity per se. Nevertheless, the progressive decline in iron levels observed with advancing COVID-19 may indicate sustained inflammatory activation and prolonged hepcidin-mediated iron sequestration. It has been observed that hospitalized patients with acute SARS-CoV-2 infection have iron deficiency or low iron concentration and transferrin saturation but high ferritin levels, while in critically ill patients the situation is reversed, with high serum iron concentration and transferrin saturation and low total iron-binding capacity [22,23]. Overall, the observed alterations in iron metabolism were associated with markers of inflammation in patients with COVID-19. While these findings may reflect interactions between immune activation and iron homeostasis, the observational nature of the study does not allow conclusions regarding the underlying mechanism. Further studies are warranted to clarify these relationships.

The next investigated parameter was serum ferritin. It is known that ferritin is both the major intracellular iron-storage protein and a well-established acute-phase protein [24]. Numerous studies have demonstrated substantially higher ferritin levels in COVID-19 patients than in healthy controls, supporting our observations of an approximately 11-fold increase. Elevated ferritin concentrations have been consistently linked to activation of the inflammatory response and cytokine-mediated dysregulation of iron homeostasis [35,42,43,44]. Ferritin emerged as a central biomarker closely associated with disease severity, inflammatory activation, and biochemical disturbances. The progressive increase in ferritin concentrations across severity categories, with significantly higher levels in critically ill patients compared to those with moderate disease, supports its role as a marker of hyperinflammation. Our findings are consistent with the reported literature data [42,44]. Serum ferritin levels have been shown to be closely associated with the severity of COVID-19 and are significantly higher (hyperferritinemia) in patients with an acute course of the disease compared to those with a moderate state [4,25,26,27]. Previous studies have demonstrated significantly higher ferritin levels in severe COVID-19 cases compared with mild disease and identified ferritin as an independent contributor to disease severity [44]. Similarly, other studies have shown increased ferritin levels in patients with severe or critical COVID-19 and in those with hypoxemia, confirming its association with disease progression [42]. Ferritin is a prognostic marker, and its high levels are observed in hospitalized patients in the critical phase of COVID-19 [28]. Moreover, in our study ferritin concentrations were significantly higher in non-survivors than in survivors, which is consistent with the association between hyperferritinemia, disease severity, and adverse clinical outcome. These findings support the role of ferritin as a marker of inflammatory burden in COVID-19. However, given the observational nature of the study, our results should be interpreted as evidence of association rather than a definitive prognostic marker. Further studies are warranted to determine the clinical utility of ferritin as a prognostic biomarker in COVID-19.

Transferrin is a primary iron transport protein and a negative acute-phase protein, often reduced in inflammatory states [45]. In contrast to the two previous tests (serum iron and ferritin), transferrin levels in the study group were significantly decreased compared to healthy individuals, both before and after treatment, which is well supported by the existing literature [19,46]. The progressive reduction in transferrin levels observed with increasing disease severity is in agreement with previous reports demonstrating significantly lower transferrin concentrations in severe and critical COVID-19, highlighting the close relationship between inflammation, impaired iron transport, and disease progression [42]. As reported in the literature, transferrin concentrations were low in COVID-19 outpatients and inpatients, and this occurred despite low levels of serum iron and low transferrin levels predicting increased inflammation [19,47]. In our study, transferrin showed inverse relationships with inflammatory indices, liver enzymes, and coagulation tests, while it correlated positively with erythrocyte-related parameters. These associations indicate that transferrin reflects both inflammatory burden and overall metabolic state. The observed negative association between transferrin concentration and disease severity supports its potential utility as an indicator of disease progression. Consistent with this observation, in our study serum transferrin levels were significantly lower in non-survivors than in survivors. This finding is in line with the recognized role of transferrin as a negative acute-phase reactant, whose concentrations decrease in response to inflammation and disease severity. Reduced transferrin levels in non-survivors may therefore reflect a more pronounced inflammatory state and greater disruption of iron homeostasis. However, given the observational design and the dynamic nature of the inflammatory response, the prognostic significance of transferrin should be interpreted with caution. These findings highlight the need for longitudinal assessment of iron-related parameters in COVID-19 to better characterize their temporal changes and potential clinical utility. Transferrin saturation was also significantly decreased in COVID-19 patients compared to healthy subjects, although it was higher among patients with cytokine storm. This parameter reflects iron immediately available for erythropoiesis [48]. Low transferrin saturation, below 20% (in our study 19.8%), is a common finding in COVID-19 patients and indicates functional iron deficiency, even in the presence of elevated ferritin [20].

The soluble transferrin receptor showed more nuanced associations. It is well established that this marker can be used to differentiate iron deficiency anemia, in which serum levels are elevated, from anemia of chronic disease, in which concentrations typically remain within the normal range [49]. In our study, sTfR concentrations did not differ significantly from those observed in healthy individuals, nor were there significant differences between patients before and after treatment. The available literature provides conflicting evidence regarding changes in the concentration of this receptor in patients with COVID-19 [39,50,51]. While some authors reported elevated sTfR levels in patients admitted to the intensive care unit (ICU) compared to healthy controls, contrary to our findings, others found no significant changes in sTfR levels during a 15–18 day ICU stay [50,51]. Similarly, no significant differences in the receptor concentrations were observed between anemic patients and healthy controls on day 5 and during the period from days 5 to 15 following hospital admission [39]. The soluble transferrin receptor showed only a trend toward increasing values with disease progression and was associated with biochemical parameters and impaired oxygenation, suggesting a link between increased cellular iron demand, erythropoietic activity, and hypoxic stress. Conversely, other studies demonstrated significantly higher levels of this receptor in patients with severe COVID-19 than in those with mild or moderate disease; however, these findings were observed two months after COVID-19 onset [32].

In turn, hepcidin, a peptide hormone produced and secreted by the liver, is a central regulator of iron homeostasis [37]. Its expression increases during the immune response, and it plays a key role in iron metabolism [33]. Our findings are in agreement with numerous studies demonstrating activation of the hepcidin–ferroportin axis during SARS-CoV-2 infection. Elevated hepcidin has been reported in hospitalized COVID-19 patients and is generally attributed to inflammation-driven stimulation, particularly via IL-6 signaling [42,43,52]. In contrast to our research, several studies have reported significantly higher hepcidin concentrations in patients with severe or critical COVID-19 than in those with mild disease [42,44]. This discrepancy may be explained by limited sample size and statistical power, heterogeneity in disease stage at sampling, treatment-related effects, or differences in severity classification. Nevertheless, a clear correlation was found between hepcidin and inflammatory markers, selected hematological parameters, and biochemical laboratory tests. Multiple studies have demonstrated positive associations between hepcidin and inflammatory mediators [41,42].

The present study has several strengths that enhance the interpretability of the findings. First, the analysis includes a broad panel of iron metabolism biomarkers reflecting the different aspects of iron metabolism (transport, storage, systemic regulation, and cellular demand). Second, the study design incorporates two clinically relevant time points, at hospital admission and during hospitalization, enabling evaluation of dynamic changes in laboratory parameters in response to disease progression and treatment. Third, the simultaneous assessment of biochemical, inflammatory, hematological, coagulation, and blood gas parameters allows for integrated analysis and interpretation of relationships between iron metabolism and multiple physiological systems.

However, several limitations should be considered when interpreting the results. First, the control group differed from the COVID-19 patients with respect to age and sex distribution, which may have influenced some parameters of iron metabolism. Second, owing to the exploratory nature of the study, detailed multivariable regression analyses and corrections for multiple comparisons were not performed. Consequently, the findings should be interpreted with caution due to the increased risk of type I error. Third, a relatively small number of patients in certain disease severity subgroups may have limited the statistical power of analyses. Fourth, the potential effects of the treatments administered during hospitalization on the analyzed laboratory parameters were not specifically assessed and therefore cannot be excluded. Finally, the lack of long-term follow-up prevented evaluation of whether the observed abnormalities persisted beyond the acute phase of SARS-CoV-2 infection.

## 5. Conclusions

In conclusion, SARS-CoV-2 infection is associated with profound and dynamic alterations in iron metabolism, characterized by inflammation-driven iron sequestration and dysregulated iron distribution, with decreased serum iron, transferrin, and TSAT and increased ferritin and hepcidin. This dysregulation may contribute to impaired hemoglobin synthesis and could play a role in the development of anemia of inflammation. Ferritin and transferrin showed the strongest association with disease severity and markers of systemic inflammation, suggesting their potential relevance in the assessment of COVID-19 patients. Ferritin concentrations tended to increase, whereas transferrin levels tended to decrease with increasing disease severity, particularly among critically ill patients. Moreover, ferritin levels were significantly higher and transferrin levels significantly lower in non-survivors than in survivors, indicating that disturbances in iron metabolism may be associated with unfavorable outcomes in COVID-19. In contrast, serum iron and transferrin saturation appeared to provide limited information regarding disease severity, while hepcidin and soluble transferrin receptor may offer complementary insight into alterations in iron metabolism associated with SARS-CoV-2 infection. Further studies are required to determine the diagnostic and prognostic value of these biomarkers.

## Figures and Tables

**Table 1:** Modified Early Warning Score (MEWS) [31].

Score	3	2	1	0	1	2	3
Respiratory rate (breaths/min)	≥25	21–24		12–20			≤8
SatO_2_ (%)	≤91	92–93	94–95	≥96			
Temperature (°C)		≥39.1	38.1–39	36.1–38	35.1–36		≤35
Systolic blood pressure (mmHg)	≥220			111–219	101–110	91–100	≤90
Heart rate (bpm)	≥131	111–130	91–110	51–90	41–50		≤40
AVPU				Alert			Verbal, pain, unresponsive

Abbreviations: SatO_2_, oxygen saturation; bpm, beats per minute; AVPU, alert, verbal, pain, unresponsive.

**Table 2:** Laboratory characteristics of the study group and the controls.

Test	Sample 1 (S1)	Sample 2 (S2)	Controls (C)	Comparisons (*p*-Value)		
				S1 vs. C	S2 vs. C	S1 vs. S2
Biochemical tests						
ALT (IU/L)	30.7 15.3–56.0	35.0 22.0–62.0	10.1 7.75–14.6	<0.001 *	<0.001 *	0.074
AST (IU/L)	42.0 23.7–71.0	33.3 20.1–50.3	19.9 17.1–23.3	<0.001 *	<0.001 *	0.005 *
GGT (IU/L)	52.0 26.0–120	75.0 31.0–150	14.0 10.0–20.0	<0.001 *	<0.001 *	0.865
LDH (IU/L)	268 204–399	182 161–253	155 139–180	<0.001 *	<0.001 *	<0.001 *
CK (IU/L)	91.0 45.5–193	32.0 23.0–66.0	89.0 67.0–138	0.997	<0.001 *	<0.001 *
Total Bilirubin (mg/dL)	0.52 0.35–0.73	0.45 0.25–0.88	0.47 0.35–0.58	0.273	0.846	0.229
Glucose (mg/dL)	109 92.0–134	103 90.0–129	86.4 78.8–91.2	<0.001 *	<0.001 *	0.964
Cholesterol (mg/dL)	142 110–173	141 119–191	197 168–225	<0.001 *	<0.001 *	0.176
Creatinine (mg/dL)	0.85 0.71–1.24	0.77 0.60–0.95	0.86 0.76–0.94	0.506	0.009 *	0.001 *
Inflammatory markers						
IL-6 (pg/mL)	42.0 19.4–127	24.6 12.8–65.9	1.50 1.50–1.82	<0.001 *	<0.001 *	0.253
IL-18 (pg/mL)	606 456–862	302 116–532	159 69–259	<0.001 *	0.027 *	<0.001 *
CRP (mg/L)	41.1 18.1–109	12.2 4.00–36.9	0.64 0.60–1.19	<0.001 *	<0.001 *	<0.001 *
Procalcitonin (ng/mL)	0.13 0.06–0.44	0.08 0.04–0.36	0.03 0.02–0.03	<0.001 *	<0.001 *	0.130
Haptoglobin (g/dL)	2.62 1.87–3.93	1.84 1.22–2.86	1.05 0.77–1.31	<0.001 *	<0.001 *	<0.001 *
Hematological parameters and coagulation tests						
Hb (g/dL)	12.9 11.2–14.0	12.6 11.0–13.8	13.7 13.2–14.8	<0.001 *	<0.001 *	0.895
Ht (%)	38.4 33.4–41.3	37.6 33.5–40.8	39.1 37.1–42.3	0.028 *	0.010 *	0.570
RBC (×10^6^/µL)	4.27 3.68–4.65	4.13 3.75–4.61	4.59 4.32–4.93	<0.001 *	<0.001 *	0.895
MCV (fL)	89.2 85.5–94	89.5 85.7–93.2	85.6 83.5–88.4	0.001 *	<0.001 *	0.041 *
WBC (×10^3^/µL)	7.2 4.85–9.20	6.95 5.18–9.90	6.49 5.52–7.26	0.202	0.201	0.047 *
PLT (×10^3^/µL)	205 163–270	254 158–345	247 213–271	0.087	0.463	0.002 *
PT (s)	13.7 12.6–16.3	14.0 13.0–16.7	12.4 12.1–13.1	<0.001 *	<0.001 *	0.850
INR	1.14 1.05–1.37	1.18 1.09–1.44	0.97 0.94–1.03	<0.001 *	<0.001 *	0.841
Fibrinogen (mg/dL)	431 308–613	315.5 179–413	294 256–342	<0.001 *	0.722	0.465
Arterial blood gas and CO-oximetry						
pH	7.457 7.426–7.484	7.459 7.436–7.488	7.405 7.375–7.425	<0.001 *	<0.001 *	0.468
PaO_2_ (mmHg)	83.6 64.0–107	82.6 65.7–107	97.0 93.0–101	0.008 *	0.007 *	0.317
PaCO_2_ (mmHg)	36.1 34.0–39.8	38.5 34.9–41.3	39.0 37.0–41.0	0.002 *	0.241	0.058
O_2_Sat (%)	96.8 93.0–98.5	96.7 94.9–98.1	97.0 97.0–99.0	0.204	0.075	0.839
BE (mEq/L)	2.50 −0.70–4.90	3.25 1.20–5.30	0.33 −0.90–1.15	0.002 *	<0.001 *	0.020 *

Data are presented as median and interquartile range (Q1–Q3). The differences between S1 and C and S2 and C estimated by Student’s *t*-test (for Hb, RBC, MCV, PLT, pO_2_) or Mann–Whitney U test (for all other tests). The differences between S1 and S2 estimated by the Wilcoxon matched-pairs test (*n* = 40 paired samples). * Significant difference at *p* < 0.05. Abbreviations: ALT, alanine aminotransferase; AST, aspartate aminotransferase; GGT, γ-glutamyltransferase; LDH, lactate dehydrogenase; CK, creatine kinase; IL-6, interleukin 6; IL-18, interleukin 18; CRP, C-reactive protein; Hb, hemoglobin; Ht, hematocrit; RBC, red blood cells; MCV, mean corpuscular volume; WBC, white blood cells; PLT, platelets; PT, prothrombin time; INR, international normalized ratio; PaO_2_, partial pressure of arterial oxygen; PaCO_2_, partial pressure of arterial carbon dioxide; O_2_Sat, oxygen saturation; BE, base excess.

**Table 3:** Laboratory markers of iron status in the study group and the controls.

Test	Sample 1 (S1)	Sample 2 (S2)	Controls (C)	Comparisons (*p*-Value)		
				S1 vs. C	S2 vs. C	S1 vs. S2
Iron (µg/dL)	42.1 30.3–65.4	58.6 44.5–106.9	128.8 104.7–180.7	<0.001 *	<0.001 *	<0.05 *
Ferritin (µg/L)	593 236–1414	672 304–1245	54.5 27.3–114.5	<0.001 *	<0.001 *	<0.05 *
Transferrin (g/L)	1.66 1.21–1.88	1.67 1.24–1.88	2.72 2.39–3.06	<0.001 *	<0.001 *	0.717
Transferrin Saturation (%)	19.8 15.0–31.6	31.3 20.7–48.6	35.0 16.4–49.9	0.048	0.994	0.076
sTfR (mg/L)	2.75 2.22–3.75	2.51 2.11–3.86	2.77 2.32–3.42	0.975	0.681	0.469
Hepcidin (pg/mL)	26,413 14,917–3,7011	26,908 16,970–41,435	4194 843–8974	<0.001 *	<0.001 *	0.295

Data are presented as median and interquartile range (Q1–Q3). The differences between S1 and C and S2 and C estimated by the Mann–Whitney U test. The differences between S1 and S2 estimated by the Wilcoxon matched-pairs test (*n* = 40 paired samples). * Significant difference at *p* < 0.05. Abbreviations: sTfR, soluble transferrin receptor.

**Table 4:** Laboratory markers of iron status in the study group according to gender.

Test	Gender		
	Female (*n* = 33)	Male (*n* = 54)	Comparisons (*p*-Value)
Iron (µg/dL)	47.1 (31.9–62.7)	39.4 (29.3–67.7)	0.899
Ferritin (µg/L)	367 (197–832)	682 (303–1755)	0.011 *
Transferrin (g/L)	1.72 (1.46–1.84)	1.50 (1.15–1.94)	0.198
Transferrin saturation (%)	19.7 (13.7–31.3)	21.0 (15.0–32.3)	0.614
sTfR (mg/L)	2.92 (2.30–4.24)	2.67 (2.11–3.43)	0.136
Hepcidin (pg/mL)	25,061 (13,831–32,058)	29,478 (15,876–40,096)	0.187

Data are presented as median and interquartile range (Q1–Q3). The difference in iron test level according to the gender of patients was estimated via the Mann–Whitney U test. * Significant difference at *p* < 0.05. Abbreviation: sTfR, soluble transferrin receptor.

**Table 5:** Laboratory markers of iron status in the study according to the clinical conditions.

COVID-19 Patients	Clinical Characteristics		
	Yes	No	Comparisons (*p*-Value)
Cytokine storm:			
Iron (µg/dL)	39.8 (27.8–76.0)	43.1 (32.0–62.7)	0.921
Transferrin (g/L)	1.20 (1.00–1.66)	1.77 (1.50–1.97)	<0.001 *
Transferrin saturation (%)	22.9 (16.7–45.9)	18.8 (13.5–29.9)	0.011 *
sTfR (mg/L)	2.72 (2.09–4.24)	2.75 (2.30–3.44)	0.704
Hepcidin (pg/mL)	28,845 (14,193–36,797)	25,913 (14,917–37,924)	0.773
Ferritin (µg/L)	1367 (532–1947)	391 (197–693)	<0.001 *
Comorbidities:			
Iron (µg/dL)	36.8 (26.0–60.9)	50.6 (35.2–67.8)	0.094
Transferrin (g/L)	1.59 (1.15–1.93)	1.73 (1.50–1.87)	0.222
Transferrin saturation (%)	19.6 (13.7–31.3)	21.0 (16.2–36.2)	0.269
sTfR (mg/L)	2.94 (2.18–4.16)	2.58 (2.25–3.02)	0.204
Hepcidin (pg/mL)	25,760 (13,831–36,664)	28,476 (18,862–39,749)	0.307
Ferritin (µg/L)	574 (230–1349)	631 (263–1549)	0.613
Vaccination:			
Iron (µg/dL)	38.0 (29.4–65.6)	43.1 (31.1–65.3)	0.809
Transferrin (g/L)	1.67 (1.25–1.83)	1.67 (1.22–1.93)	0.967
Transferrin saturation (%)	21.0 (14.7–36.2)	19.7 (14.9–28.9)	0.796
sTfR (mg/L)	2.61 (2.18–3.65)	2.79 (2.30–3.80)	0.835
Hepcidin (pg/mL)	21,435 (13,591–33,838)	28,106 (17,360–38,294)	0.169
Ferritin (µg/L)	579 (197–1301)	606 (277–1417)	0.451
Surviving/non-surviving:	Survivors	Non-survivors	
Iron (µg/dL)	47.0 (31.9–67.7)	34.2 (22.5–58.5)	0.058
Transferrin (g/L)	1.75 (1.41–1.95)	1.15 (0.83–1.50)	<0.001 *
Transferrin saturation (%)	19.7 (14.8–31.5)	22.5 (15.3–32.3)	0.669
sTfR (mg/L)	2.65 (2.30–3.43)	2.98 (1.71–4.37)	0.631
Hepcidin (pg/mL)	26,066 (14,193–37,924)	27,780 (21,410–36,797)	0.552
Ferritin (µg/L)	523 (230–1225)	1271 (511–2550)	0.015 *

Data are presented as median and interquartile range (Q1–Q3). The differences in the levels of iron markers between different groups of patients were estimated via the Mann–Whitney U test. * Significant difference at *p* < 0.05. Abbreviation: sTfR, soluble transferrin receptor.

**Table 6:** Serum iron status test levels according to COVID-19 severity.

COVID-19 Severity	Iron (µg/dL)	Ferritin (µg/L)	Transferrin (g/L)	Transferrin Saturation (%)	sTfR (mg/L)	Hepcidin (pg/mL)
Moderate (*n* = 58)	47.2	391	1.75	20.9	2.63	24,819
	33.4–67.2	214–672	1.43–1.95	14.7–31.3	2.24–3.32	13,892–35,034
Severe (*n* = 13)	36.8	1176	1.43	19.3	3.00	32,451
	24.2–57.2	535–1519	1.07–1.76	14.5–37.7	2.11–4.23	13,831–38,403
	^1^ *p* = 0.408	^1^ *p* = 0.081	^1^ *p* = 0.142	^1^ *p* = 1.000	^1^ *p* = 1.000	^1^ *p* = 0.665
Critical (*n* = 16)	34.1	1394	1.16	19.8	4.12	29,452
	25.5–52.3	762–2401	0.94–1.51	15.3–39.5	2.08–4.79	26,107–43,569
	^1^ *p* = 0.330	^1^ *p* = 0.005 *	^1^ *p* < 0.001 *	^1^ *p* = 1.000	^1^ *p* = 0.301	^1^ *p* = 0.129
	^2^ *p* = 1.000	^2^ *p* = 1.000	^2^ *p* = 0.236	^2^ *p* = 1.000	^2^ *p* = 0.912	^2^ *p* = 1.000

Data are presented as median and interquartile range (Q1–Q3). The values of iron, ferritin, transferrin, transferrin saturation, sTfR, and hepcidin differed depending on the degree of disease severity as determined by the Kruskal–Wallis test (H = 3.981, *p* = 0.137) (H = 12.3, *p* = 0.002) (H = 19.0, *p* < 0.001) (H = 2.0, *p* = 0.887) (H = 2.70, *p* = 0.259) (H = 4.80, *p* = 0.090) followed by a further post hoc analysis. * Significant difference at *p* < 0.05. ^1^ in comparison to the moderate stage. ^2^ in comparison to the severe stage. Abbreviations: sTfR, soluble transferrin receptor.

**Table 7:** The association between iron, ferritin, and transferrin and clinical data in COVID-19 patients.

Test	Iron		Ferritin		Transferrin	
	R	*p*	R	*p*	R	*p*
Age	−0.089	0.386	0.198	0.053	−0.161	0.117
Disease severity	0.011	0.912	0.044	0.668	−0.210	0.040
Cytokine storm	−0.011	0.918	0.354	<0.001	−0.473	<0.001
Comorbidities	0.172	0.093	0.052	0.613	0.126	0.222
Vaccination	−0.025	0.897	−0.079	0.451	0.005	0.964

R, Spearman’s rank correlation coefficient. *p* < 0.05 significant correlation.

**Table 8:** The association between iron, ferritin, and transferrin and laboratory tests in COVID-19 patients.

Test	Iron		Ferritin		Transferrin	
	R	*p*	R	*p*	R	*p*
ALT (IU/L)	0.115	0.265	0.345	<0.001	−0.142	0.167
AST (IU/L)	0.167	0.105	0.449	<0.001	−0.270	0.008
GGT (IU/L)	0.080	0.448	0.258	0.012	−0.083	0.430
LDH (IU/L)	0.151	0.147	0.488	<0.001	−0.320	0.002
CK (IU/L)	0.112	0.288	0.332	0.001	−0.085	0.415
Total Bilirubin (mg/dL)	0.212	0.039	0.310	0.002	−0.135	0.192
Glucose (mg/dL)	0.129	0.220	0.231	0.027	−0.033	0.753
Cholesterol (mg/dL)	0.178	0.083	−0.087	0.398	0.391	<0.001
Creatinine (mg/dL)	0.025	0.805	0.242	0.018	−0.007	0.947
IL-6 (pg/mL)	0.026	0.805	0.471	<0.001	−0.500	0.000
IL-18 (pg/mL)	0.048	0.642	0.544	0.000 *	−0.510	0.000
CRP (mg/L)	−0.135	0.190	0.503	0.000	−0.403	<0.001
Procalcitonin (ng/mL)	−0.094	0.365	0.451	<0.001	−0.431	<0.001
TNF-α	0.033	0.745	0.330	<0.001	−0.190	0.063
IFN-γ	−0.072	0.484	0.411	0.000	−0.193	0.058
Hb (g/dL)	0.116	0.258	0.236	0.021	0.216	0.035
Ht (%)	0.116	0.260	0.177	0.085	0.230	0.024
RBC (×10^6^/µL)	0.034	0.745	0.098	0.340	0.283	0.005
MCV (fL)	0.177	0.083	0.153	0.136	−0.241	0.018
WBC (×10^3^/µL)	−0.238	0.020	0.158	0.124	−0.310	0.002
PLT (×10^3^/µL)	−0.039	0.707	−0.098	0.342	0.139	0.176
PT (s)	0.029	0.786	0.127	0.240	−0.229	0.029
INR	0.031	0.773	0.128	0.227	−0.232	0.028
Fibrinogen (mg/dL)	−0.090	0.415	0.351	<0.001	−0.127	0.244
pH	−0.173	0.096	0.024	0.821	−0.173	0.096
PaO_2_ (mmHg)	−0.099	0.359	−0.039	0.721	0.138	0.203
PaCO_2_ (mmHg)	0.120	0.250	−0.156	0.136	0.124	0.237
O_2_Sat (%)	−0.010	0.925	−0.098	0.348	0.137	0.191
BE (mEq/L)	0.064	0.537	−0.120	0.252	−0.028	0.789

R, Spearman’s rank correlation coefficient. * *p* < 0.05 significant correlation. Abbreviations: ALT, alanine aminotransferase; AST, aspartate aminotransferase; GGT, γ-glutamyltransferase; LDH, lactate dehydrogenase; CK, creatine kinase; IL-6, interleukin 6; IL-18, interleukin 18; CRP, C-reactive protein; TNF-α, tumor necrosis factor alpha; IFN-γ, interferon gamma; Hb, hemoglobin; Ht, hematocrit; RBC, red blood cells; MCV, mean corpuscular volume; WBC, white blood cells; PLT, platelets; PT, prothrombin time; INR, international normalized ratio; PaO_2_, partial pressure of arterial oxygen; PaCO_2_, partial pressure of arterial carbon dioxide; O_2_Sat, oxygen saturation; BE, base excess.

**Table 9:** The association between transferrin saturation, sTfR, and hepcidin and clinical data in COVID-19 patients.

Test	Transferrin Saturation		sTfR		Hepcidin	
	R	*p*	R	*p*	R	*p*
Age	−0.005	0.958	0.073	0.480	0.149	0.163
Disease severity	0.042	0.687	0.170	0.098	0.054	0.616
Cytokine storm	0.264	0.011	−0.039	0.704	0.031	0.771
Comorbidities	0.116	0.269	−0.130	0.205	0.109	0.308
Vaccination	0.028	0.794	−0.022	0.833	−0.150	0.169

R, Spearman’s rank correlation coefficient. *p* < 0.05 significant correlation.

**Table 10:** The association between transferrin saturation, sTfR, and hepcidin and laboratory tests in COVID-19 patients.

Test	Transferrin Saturation		sTfR		Hepcidin	
	R	*p*	R	*p*	R	*p*
ALT (IU/L)	0.061	0.558	0.097	0.348	−0.033	0.347
AST (IU/L)	0.203	0.051	0.158	0.123	0.048	0.123
GGT (IU/L)	0.091	0.391	0.205	0.048	−0.002	0.048
LDH (IU/L)	0.253	0.015	0.087	0.402	0.241	0.402
CK (IU/L)	0.101	0.345	−0.116	0.271	0.343	0.271
Total Bilirubin (mg/dL)	0.147	0.161	0.281	0.006	−0.052	0.006
Glucose (mg/dL)	0.066	0.534	0.251	0.016	0.022	0.016
Cholesterol (mg/dL)	−0.061	0.558	0.137	0.183	−0.002	0.183
Creatinine (mg/dL)	−0.031	0.765	0.045	0.664	0.219	0.039
IL-6 (pg/mL)	0.314	0.002	−0.003	0.980	0.022	0.839
IL-18 (pg/mL)	0.229	0.027	0.068	0.509	0.225	0.034
CRP (mg/L)	0.129	0.217	−0.061	0.553	0.270	0.010
Procalcitonin (ng/mL)	0.165	0.113	0.045	0.666	0.142	0.667
TNF-α	0.118	0.258	0.080	0.322	0.230	0.030
IFN-γ	0.013	0.899	0.017	0.840	0.241	0.023
Hb (g/dL)	−0.009	0.932	−0.058	0.573	0.323	0.002
Ht (%)	−0.026	0.802	−0.032	0.754	0.303	0.004
RBC (×10^6^/µL)	−0.154	0.141	0.022	0.833	0.280	0.008
MCV (fL)	0.347	<0.001	−0.005	0.958	−0.019	0.862
WBC (×10^3^/µL)	−0.096	0.360	−0.012	0.905	0.141	0.188
PLT (×10^3^/µL)	−0.109	0.300	−0.083	0.419	−0.075	0.484
PT (s)	0.084	0.438	0.225	0.033	−0.060	0.585
INR	0.087	0.423	0.224	0.034	−0.060	0.587
Fibrinogen (mg/dL)	0.080	0.474	−0.024	0.826	0.238	0.036
pH	−0.017	0.870	−0.111	0.291	−0.125	0.248
PaO_2_ (mmHg)	−0.132	0.231	−0.228	0.033	0.169	0.130
PaCO_2_ (mmHg)	0.101	0.345	−0.011	0.920	−0.068	0.532
O_2_Sat (%)	−0.016	0.878	−0.204	0.049	0.083	0.446
BE (mEq/L)	0.159	0.134	−0.142	0.174	−0.147	0.173

R, Spearman’s rank correlation coefficient. *p* < 0.05 significant correlation. Abbreviations: sTfR, soluble transferrin receptor; ALT, alanine aminotransferase; AST, aspartate aminotransferase; GGT, γ-glutamyltransferase; LDH, lactate dehydrogenase; CK, creatine kinase; IL-6, interleukin 6; IL-18, interleukin 18; CRP, C-reactive protein; TNF-α, tumor necrosis factor alpha; INF-γ, interferon gamma; Hb, hemoglobin; Ht, hematocrit; RBC, red blood cells; MCV, mean corpuscular volume; WBC, white blood cells; PLT, platelets; PT, prothrombin time; INR, international normalized ratio; PaO_2_, partial pressure of arterial oxygen; PaCO_2_, partial pressure of arterial carbon dioxide; O_2_Sat, oxygen saturation; BE, base excess.

## Data Availability

The original contributions presented in this study are included in the article. Further inquiries can be directed to the corresponding author.
